# The differential expression of PilY1 proteins by the HsfBA phosphorelay allows twitching motility in the absence of exopolysaccharides

**DOI:** 10.1371/journal.pgen.1010188

**Published:** 2022-04-29

**Authors:** Shuanghong Xue, Romain Mercier, Annick Guiseppi, Artemis Kosta, Rossella De Cegli, Severine Gagnot, Tâm Mignot, Emilia M. F. Mauriello

**Affiliations:** 1 Laboratoire de Chimie Bactérienne, CNRS, Aix-Marseille Univ, Marseille, France; 2 Microscopy Platform, Institut de Microbiologie de la Méditerranée, CNRS, Marseille, France; 3 Telethon Institute of Genetics and Medicine (TIGEM), Naples, Italie; Michigan State University, UNITED STATES

## Abstract

Type Four Pili (T4P) are extracellular appendages mediating several bacterial functions such as motility, biofilm formation and infection. The ability to adhere to substrates is essential for all these functions. In *Myxococcus xanthus*, during twitching motility, the binding of polar T4P to exopolysaccharides (EPS), induces pilus retraction and the forward cell movement. EPS are produced, secreted and weakly associated to the *M*. *xanthus* cell surface or deposited on the substrate. In this study, a genetic screen allowed us to identify two factors involved in EPS-independent T4P-dependent twitching motility: the PilY1.1 protein and the HsfBA phosphorelay. Transcriptomic analyses show that HsfBA differentially regulates the expression of PilY1 proteins and that the down-regulation of *pilY1*.*1* together with the accumulation of its homologue *pilY1*.*3*, allows twitching motility in the absence of EPS. The genetic and bioinformatic dissection of the PilY1.1 domains shows that PilY1.1 might be a bi-functional protein with a role in priming T4P extension mediated by its conserved N-terminal domain and roles in EPS-dependent motility mediated by an N-terminal DUF4114 domain activated upon binding to Ca^2+^. We speculate that the differential transcriptional regulation of PilY1 homologs by HsfBA in response to unknown signals, might allow accessorizing T4P tips with different modules allowing twitching motility in the presence of alternative substrates and environmental conditions.

## Author summary

In the motile bacterium *Myxococcus xanthus*, T4P mediate twitching motility by binding to the sugar moiety of the extracellular matrix deposited on the neighboring cells or on the substrate. The binding of T4P to these sugars also termed exopolysaccharides (EPS) stimulates the pilus fiber retraction pulling the cell forwards. In this study, we performed a series of genetic analyses leading to the discovery that *M*. *xanthus* cells can move in the absence of EPS if two conditions are fulfilled: the *pilY1*.*1* gene is down-regulated and the PilY1.3 protein is accumulated on pili. RNAseq, qRT-PCR and gel retardation assays show that the differential accumulation of PilY1 proteins is under the control of the HsfBA phosphorelay, which up-regulates the expression of *pilY1*.*1* and down-regulates that of a homologue, *pilY1*.*3*. We also found that PilY1.1 has a domain at the N terminus probably requiring Ca^2+^ to be active in EPS-dependent motility, and a conserved domain at the C terminus essential for T4P assembly. Conversely, PilY1.3 contains a Von Willebrand factor A (VWA) domain and is potentially involved in the binding to proteins. We speculate that thanks to the HsfBA regulation, T4P can be equipped with different PilY1 homologues to allow twitching motility in the presence of different substrates.

## Introduction

Type Four Pili (T4P) are widespread bacterial and archaeal appendages responsible for diverse processes such as motility, uptake of exogenous genetic material and adherence to host cells during infections [1]. While these T4P-mediated processes largely differ relative to their function, they share the same operating principle based on finely regulated cycles of extension and retraction of the pilus fibers [2–4]: T4P extend, bind to a substrate, which in turn induces retraction. These cycles allow to pull the cell body forwards (motility) [5], bring DNA fragments near the cell envelop for internalization (competence) [6] and favor the invasion of a host cell by a bacterium (infection) [7]. During retraction a force higher than 100 pN is generated [4,8], making T4P the strongest known nanomachine. T4P are also evolutionary related to Type II Secretion and archaeal flagellum systems. Binding to substrates can take place through major pilins which in some cases possess a bimodular organization [9], minor pilins such as PilV from *Neisseria* [10] or other pilus proteins like PilY1 from *Pseudomonas aeruginosa* [11].

T4P are composed of a basal body spanning the cell envelope and a fiber of a few microns long extending outside the cell. Here, we will describe the T4P protein content using the nomenclature from *Myxococcus xanthus*. The extracellular fiber is composed of PilA, the major pilin. The basal body is composed of an outer membrane pore complex, an alignment complex and a motor complex [12]. The outer membrane pore complex contains the secretin PilQ and peptidoglycan binding protein TsaP. The alignment complex spans the periplasm and is made of the PilM, PilN, PilO and PilP structural components. The motor complex includes PilC, forming a ring in the inner membrane, PilB and PilT. PilB and PilT are the ATPases powering the extension and retraction of T4P, respectively [13]. T4P also need a priming complex composed of PilY1 and a series of minor pilins including FimU, PilW, PilX and PilV. This complex is found in the periplasmic part of the basal body, probably to prime T4P assembly, but also at the tip of the pili fiber, possibly to mediate the pilus adherence to substrates [14]. In fact, it has been shown that *P*. *aeruginosa* PilY1 contains an integrin binding arginine-glycine-aspartic acid (RGD) motif playing important roles in the adhesion to epithelial host cells [11].

*M*. *xanthus* is a Gram-negative deltaproteobacterium which can feed on bacterial preys while moving on surfaces in swarms or as individual cells. The absence of nutrients induces cells to aggregate into mounds later maturing into fruiting bodies. Inside these multicellular structures, bacteria differentiate into metabolically quiescent and heat-resistant spores [15]. *M*. *xanthus* cells can move by the aid of two motility systems, each employing a distinct nanomachine. Gliding motility requires the presence of focal adhesion Agl/Glt complexes that span the cell envelop and are characterized by the absence of extracellular appendages [16]. Cells move by gliding preferentially on hard surfaces (1.5% agar) and as individuals. For this, researchers often refer to Adventurous (A) motility to describe this type of motility. On softer surfaces (0.5% agar), cells preferentially move as large swarms by twitching using T4P. For this reason, twitching is also termed Social (S) motility. During twitching motility, T4P bind to exopolysaccharides composing the extracellular matrix surrounding a neighboring cell or deposited on the substrate as cells move forward. Binding to exopolysaccharides induces the retraction of T4P, which allows the forward movement of the cell body [5]. *M*. *xanthus* produces three different exopolysaccharides: a main exopolysaccharide (EPS), a biosurfactant polysaccharide (BPS) and a polysaccharide composing the spore coat (MASC), each synthesized and secreted by a genetically distinct pathway [17]. While both exo-sugars might have a role in twitching [17], the absence of EPS completely abolishes T4P-mediated motility, suggesting a major role of this sugar for this type of motility.

Interestingly and uniquely, EPS production is regulated by a chemosensory system, Dif, in *M*. *xanthus* [18–20]. The Dif pathway contains core homologues of Che proteins: a methyl-accepting chemotaxis-like protein (DifA), a CheA histidine kinase (DifE), a CheW docking protein (DifC) and a CheY response regulator (EpsW). DifA, DifE, DifC and EpsW positively regulate EPS production through the DifE kinase activity on EpsW [21]. Previous data suggest that EpsW might directly activate the WzxX/WzyX dependent pathway for EPS production by yet unknown mechanisms [21].

In order to find the EpsW interacting partners for the activation of EPS biosynthesis and export in *M*. *xanthus*, we used a genetic screen. Starting with a *ΔepsW* background with no EPS and, thus, no twitching motility, we searched for suppressors with restored EPS and motility. We reasoned that potential mutations that restored EPS would constitutively activate proteins otherwise requiring an interaction with EpsW to function. The result of our genetic screen was unexpected as it brought to light suppressors with restored twitching motility but not EPS production. In these suppressors, T4P could, thus, promote motility in the absence of EPS. Mutations were located in two main loci: a locus containing *pilY1*.*1*, encoding a protein previously shown to prime T4P assembly in *M*. *xanthus* [14] and the *hsfBA* operon. HsfBA is a phosphorelay where a hybrid histidine kinase, HsfB, phosphorylates the NtrC-like response regulator HsfA to directly regulate the transcription of a heat-shock gene (*lonD*) [22] and genes involved in the production of secondary metabolites such as DKxanthene (*dkxC*), myxovirescin (*taA*) and myxochromide (*mxan4077*) [23]. HsfA-regulated genes might have roles in *M*. *xanthus* development, as the insertion of an antibiotic cassette in *hsfA* delays fruiting body formation [23]. So far, there are no reports on the role of HsfBA in *M*. *xanthus* motility.

In this work, we show that HsfBA is directly involved in modulating the expression of *pilY1*.*1* and *pilY1*.*3* and that the absence or down-regulation of *pilY1*.*1* together with the accumulation of PilY1.3 on the pilus fibers allows motility in the absence of EPS. We also show that while the N-terminal domain of PilY1.1 is involved in EPS-dependent motility, potentially upon activation by Ca^2+^ binding, its conserved C-terminal is essential for priming pilus assembly. Instead, PilY1.3 possesses a von Willebrand domain at the N terminus, whereas the C terminus shows sequence homology and redundant functions with that of PilY1.1. We speculate that, thanks to a differential transcriptional regulation by HsfBA, PilY1.1 and PilY1.3 might decorate the pilus fiber to allow adhesion to different components of the extracellular matrix.

## Results

### Null point mutations in a two-component system and a pilus assembly operon restore Type IV Pilus-mediated motility in the absence of EPS

**Fig 1:**
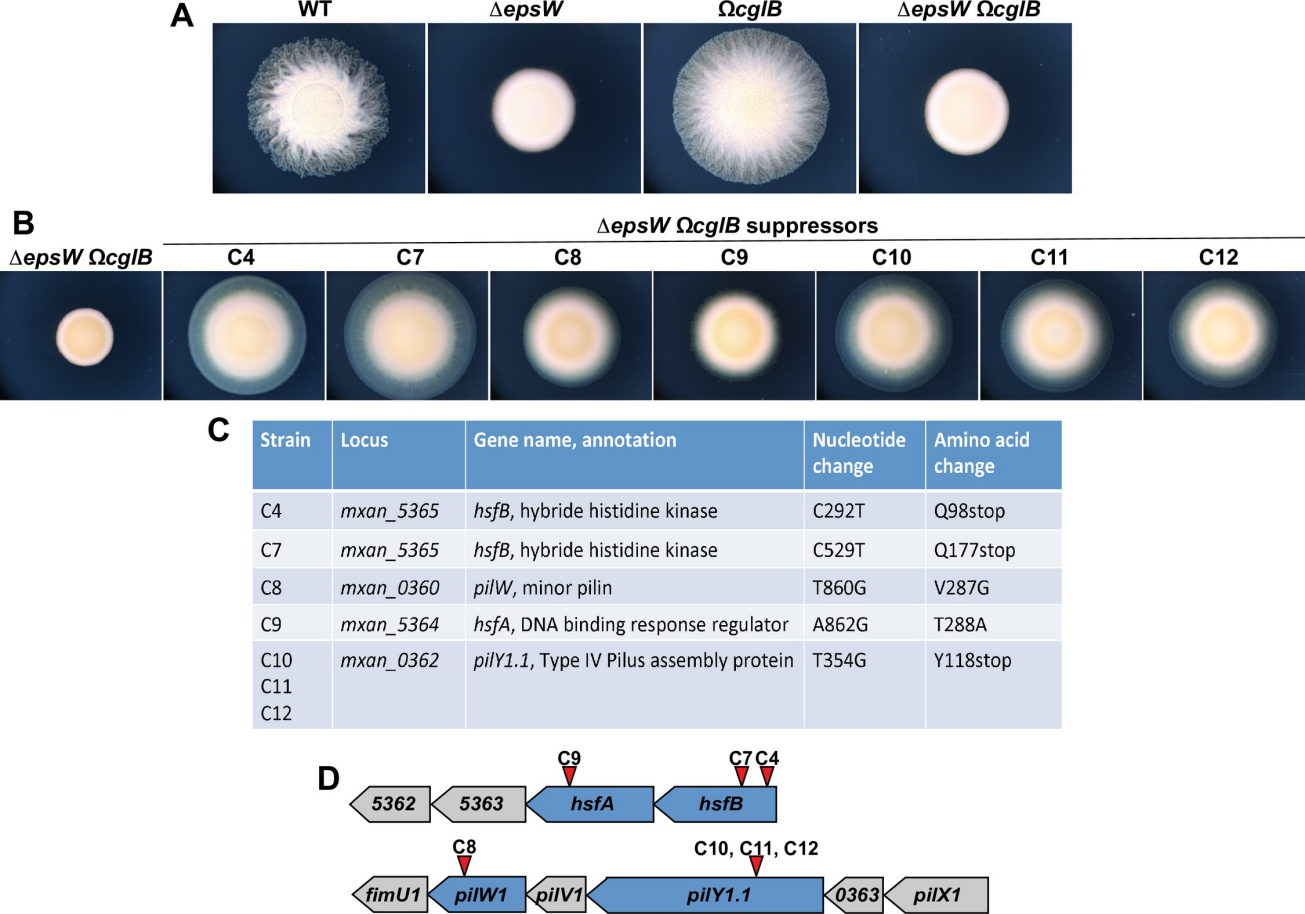
Seven suppressor mutants restore motility of a *M*. *xanthus ΔepsW ΩcglB* strain. (A) Motility phenotypic assays of DZ2 (wild type), EM650 (*ΔepsW*), TM770 (*ΩcglB*), EM749 (*ΔepsW ΩcglB*), EM785 (*ΔepsW ΩcglB sup4*), EM788 (*ΔepsW ΩcglB sup7*), EM789 (*ΔepsW ΩcglB sup8*), EM790 (*ΔepsW ΩcglB sup9*), EM791 (*ΔepsW ΩcglB sup10*), EM792 (*ΔepsW ΩcglB sup11*), EM793 (*ΔepsW ΩcglB sup12*), on 0.5% agar and imaged at 48H (B) Motility phenotypic assays of seven suppressor mutants on 0.5% agar and imaged at 72H. (C) List of seven suppressor mutations. (D) Schematic representation of the *M*. *xanthus* genomic region containing the *mxan_5362–5366* genes and *mxan_0359–0364* genes. Red arrows represent the mutation positions.

In order to find the EpsW interacting partners for the activation of the EPS biosynthetic pathway, we performed a genetic screen using a *ΔepsW ΩcglB* strain lacking EpsW and CglB [24], a long-known gliding motility protein [25]. As previously [26], we took advantage of the fact that this strain lacks both twitching (because of the absence of EPS, *ΔepsW*) and gliding motility (*ΩcglB*) (Fig 1A). Although erratic movements can be observed in *ΔepsW ΩcglB* cell groups, due to the fact that T4P are still active in the absence of *epsW*, these movements do not produce net movement (S1 Movie) and are lost in the absence of the major pilin PilA (S2 Movie). Starting with a *ΔepsW ΩcglB* genetic background, suppressors with restored motility could be easily isolated from swarms appearing at the edge of the parental non-swarming colony spotted on agar plates. We, thus, selected several mutants with restored motility (Fig 1B and S3–S7 Movies) and sequenced them by whole-genome sequencing. Mutations mapped in two loci: *mxan_5362-mxan_5365*, coding for a the HsfA-HsfB two component system (Volz *et al*., 2012) and *mxan_0359-mxan_0364*, coding for pilus assembly proteins [14]. Three point mutants generated stop codons in either HsfB (Q98stop or Q177stop) or PilY1.1 (Y118stop). Two others led to one amino acid substitution in either HsfA (T288A) or PilW1 (V287G) (Fig 1C and 1D).

**Fig 2:**
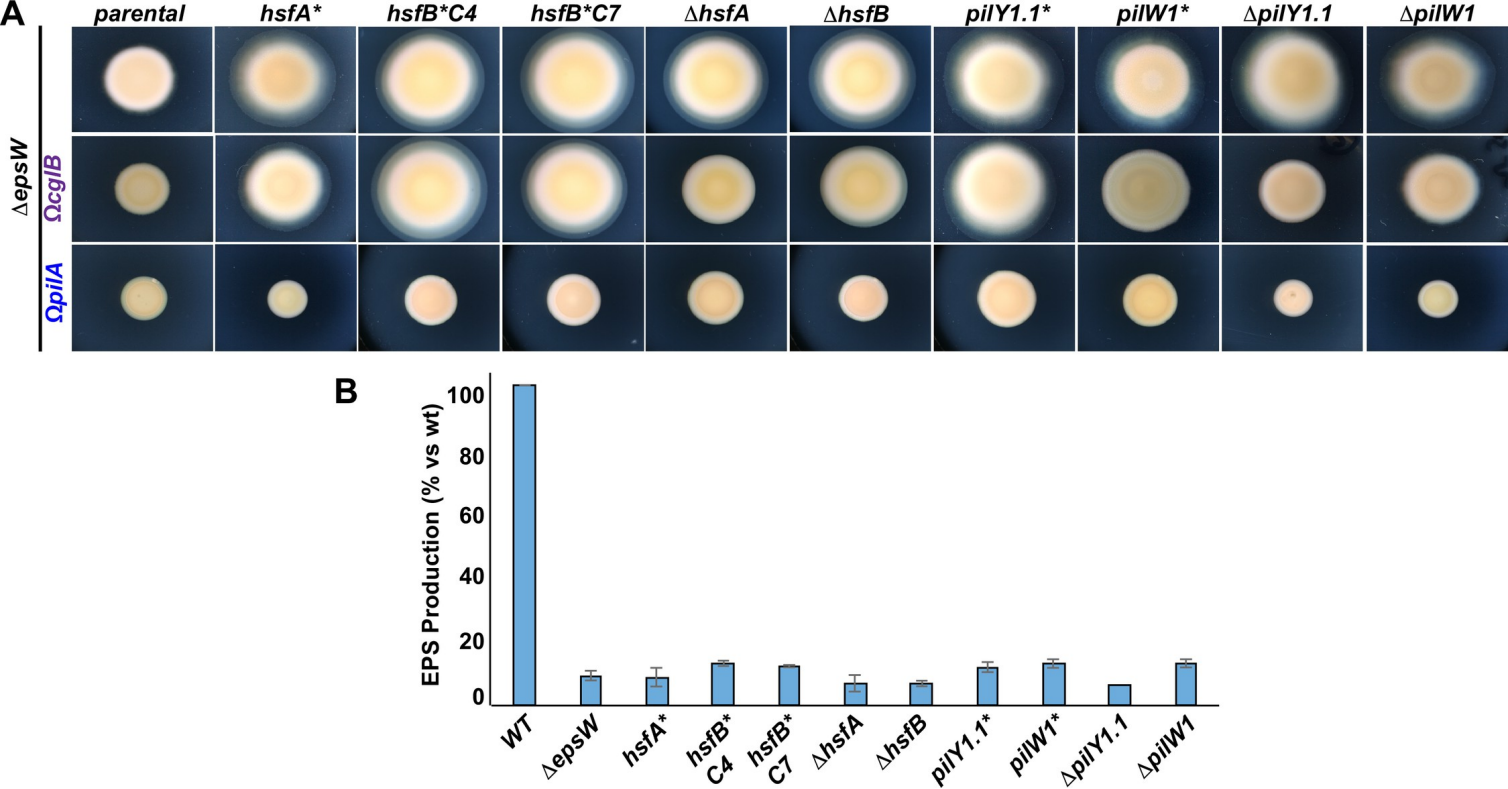
Δ*epsW* suppressors are loss-of-function mutants and their motility restoration is T4P dependent and EPS-independent. (A) Motility assays of backcrossed suppressors and in-frame deletion mutants in Δ*epsW*, Δ*epsWΩcglB* and Δ*epsWΩpilA* backgrounds on 0.5% agar at 72H. hsfA* corresponds to suppressor C9, hsfB*C4 to C4, hsfB*C7 to C7, *pilY1*.1* to C10/C11/C12, pilW1* to C8. (B) Trypan Blue assays showing the relative EPS production of the indicated strains. All data represents the mean and standard deviation of at least three biological replicates. Raw data are available in S4 Table.

The alleles identified from the suppressor screen were reconstructed, cloned into appropriate vectors and each reinserted into the *ΔepsW* genome at the endogenous locus as replacement of the respective wild type gene. The resulting backcrossed strains all showed restored motility as the originally isolated strains, confirming that the observed phenotypes were not due to the accumulation of multiple mutations (Fig 2A). Deletion mutants in *hsfA*, *hsfB*, *pilY1*.*1* and *pilW1* were also constructed and showed phenotypes similar to those observed for the respective point mutants (Fig 2A). This suggests that all selected point mutations, including those generating amino acid substitutions, abolished the gene functions.

Then, we wanted to verify that the observed phenotypes were indeed due to a restoration of EPS production. For this, we performed standard colorimetric Trypan Blue assays allowing the detection of EPS on cells. To our surprise, results show that none of the strains produced significant amounts of EPS (Fig 2B). For this, we concluded that the observed motility could be T4P-dependent but EPS-independent.

To further verify that the restored motility was twitching motility, we systematically inserted *ΩcglB* [24] *or ΩpilA* [27] (as mentioned above, *cglB* codes for a gliding motility protein and *pilA* for the T4P major pilin) in all our deletion and backcrossed point mutants (Fig 2A). While *ΩcglB* led to a reduction of the colony diameter as compared to the parental strains (probably due to the contribution of A motility to the total swarming diameters of colonies expressing *cglB*), only *ΩpilA* completely abolished motility in all strains. These results show that deletions of *hsfAB*, *pilY1*.*1* or *pilW1* restore T4P-mediated motility in the absence of EPS.

### The HsfA-HsfB phosphorelay divergently regulates the expression of *pilY1*.*1* and *pilY1*.*3*

**Fig 3:**
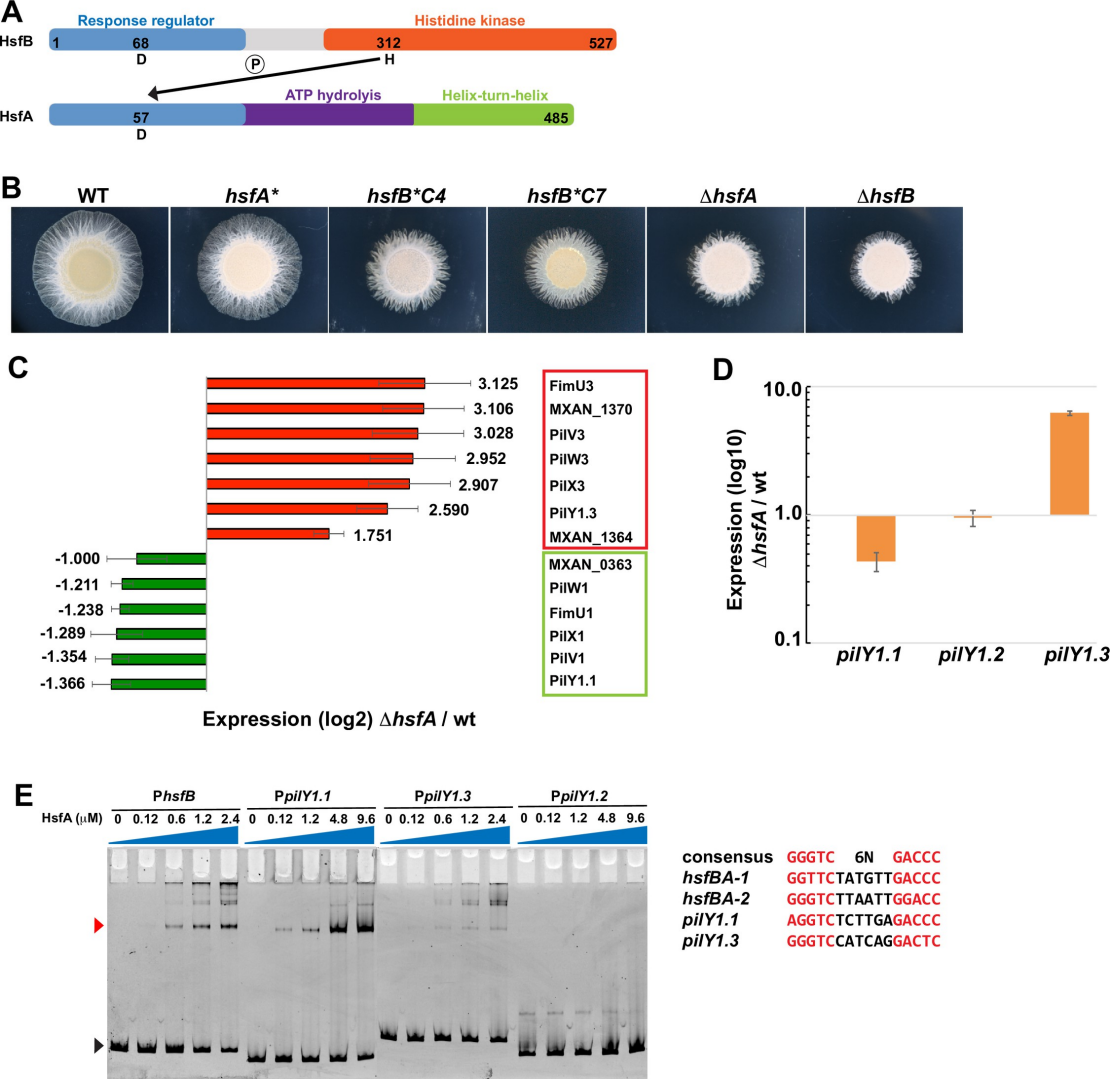
*pilY1*.*1* and *pilY1*.*3* are divergently regulated by the HsfBA phosphorelay. (A) Schematic representation of HsfB and HsfA. Numbers indicate the aminoacid position; the response regulator domains are in blue; the histidine kinase domain in orange; the ATP hydrolysis domain in purple; the helix-turn-helix domain in green. The arrow indicates the phosphotransfer from the conserved histidine H in HsfB to the aspartate D in HsfA. (B) Motility phenotypes of DZ2 (wild type), EM817 (hsfA* from sup9), EM799 (hsfB*C4 from sup4), EM816 (hsfB*C7 from sup7), EM806 (*ΔhsfA*) and EM807 (*ΔhsfB*) on 0.5% agar at 48H. The star * represents suppressor mutations. (C) Relative expression of the indicated genes in the Δ*hsfA* mutant measured as log2-fold changes by RNA-Seq. Results are mean values from three biological replicates. The green and red colors indicate up- and down-regulation, respectively, as compared to wild type. (D) Relative expression of the indicated genes in the Δ*hsfA* mutant vs wild type measured as log10-transformed fold changes by qRT-PCR. Results are mean values from two biological replicates. Error bars indicate standard deviations. Raw data are available in S4 Table. (E) Representative Electrophoretic Mobility Shift Assays (EMSA) on 6.5% polyacrylamide. The indicated concentrations of purified His_6_-HsfA were incubated with the indicated DNA fragments. White and red arrowheads indicate free DNA and His_6_-HsfA-bound DNA, respectively. Putative HsfA binding in P_*hsfB*_, P_*pilY1*.*1*_, P_*pilY1*.*3*_ are aligned against the consensus. Conserved nucleotides are in red.

Deletions in *hsfA*, *hsfB*, *pilY1*.*1* or *pilW1* were all able to restore twitching motility in the absence of EPS (Fig 2A). This suggests that these proteins might all be involved in the same pilus function. We first focused on *hsfA* and *hsfB*. These genes are part of the *hsf* operon encoding a phosphorelay including HsfB, a histidine kinase bearing a response regulator domain at the N terminus, an unusual architecture for a hybrid kinase; and HsfA, a transcriptional regulator with a response regulator domain, likely the final acceptor of the phosphoryl group in the phosphorelay, and a DNA binding domain [22] (Fig 3A). Interestingly, while *hsfA* and *hsfB* deletions allow twitching motility in the absence of EPS (Fig 2A), the same deletions interfere negatively with motility in the presence of EPS (Fig 3B).

To better understand the function of the HsfBA phosphorelay in twitching and its link with PilY1.1 and PilW1, we characterized the HsfA regulon by RNAseq. RNA was extracted, and Illumina-based deep sequencing generated an average of 20.6 million reads from wild type cells and 21.4 million from *ΔhsfA* cells. The experiment was conducted in three biological replicates. An average of 86.5% of reads in each library mapped to the *M*. *xanthus* genome. The gene expression profile in both strains was determined, and genes with a greater than 3 log_2_- fold changes in expression are presented in Figs 3C and S1. As shown in the plots from Figs 3C and S1, HsfA regulates two pilus operons, one containing *pilY1*.*1* and the other one *pilY1*.*3* (these operons will be further described below); eight predicted signaling genes; 21 genes encoding enzymes; three metabolic pathways; six genes encoding lipoproteins; three genes involved in iron uptake and 12 genes of unknown functions (Figs 3C, S1 and S1 Table). While we cannot rule out the possibility that these genes are indirectly involved in twitching motility, the only loci with an obvious function in twitching motility in the HsfA regulon are those containing *pilY1*.*1* and *pilY1*.*3* (Figs 3C, S1 and S1 Table). This result suggests that the effects of the HsfBA phosphorelay on twitching are likely exerted through PilY1 proteins and their associated minor pilins.

Interestingly, while only *pilY1*.*1* was hit in our initial genetic screen, *pilY1*.*1* and *pilY1*.*3* are both regulated by HsfA: *pilY1*.*1* is down-regulated in *ΔhsfA*, consistently with the fact that the *ΔhsfA* and *ΔpilY1*.*1* strains have similar suppression phenotypes (Fig 2A); *pilY1*.*3* is, instead, up-regulated in *ΔhsfA*. BLAST analyses show that *M*. *xanthus* carries a third homologue of PilY1.1 and PilY1.3, namely PilY1.2. The *pilY1*.*2* gene does not appear to be part of the HsfA regulon.

In order to verify these regulations, we performed qRT-PCR to quantify the expression of *pilY1*.*1*, *pilY1*.*2* and *pilY1*.*3* in cells lacking HsfA as compared to wild type. Results show that the regulation of these genes follows the same trend as that observed by RNAseq with the expression of *pilY1*.*1* reduced by 2.5 folds in *ΔhsfA* as compared to wild type and that of *pilY1*.*3* being increased by 6.4 folds (Fig 3D). The expression of *pilY1*.*2* was unchanged.

Finally, to further confirm that HsfBA regulates *pilY1*.*1* and *pilY1*.*3* but not *pilY1*.*2*, we searched for an HsfA binding box in the promoter regions of these genes [23]. While we found regions similar to the HsfA consensus sequence in the *pilY1*.*1* and *pilY1*.*3* promoters, we could not find an HsfA binding box in the *pilY1*.*2* promoter (Fig 3E).

To verify that HsfA directly binds these promoter regions, we performed gel retardation assays. For this, we heterologously produced and purified HsfA fused to a 6-Histidine tag (His_6_-HsfA) (S2 Fig) and mixed it with approximately 150 bp DNA fragments including each a *pilY1*.*1*, *pilY1*.*2* or *pilY1*.*3* promoter regulatory region. We then loaded the different mixes on acrylamide gels and, as shown in Fig 3E, while the migration of the bands corresponding to *pilY1*.*1* and *pilY1*.*3* was retarded in the presence of HsfA, the migration of *pilY1*.*2* remained unchanged. This result confirms that HsfA directly binds the promoter regions of *pilY1*.*1* and *pilY1*.*3* but not that of *pilY1*.*2*. The presence of unlabeled specific DNA significantly reduced the shift of *pilY1*.*2* and *pilY1*.*3* promoter regions (S3A Fig). Conversely, the addition of non-specific poly dI-dC did not change the amounts of shifted fragments (S3B Fig). Altogether these results suggest that in absence of HfsA the expression of *pilY1*.*1* is reduced while that of *pilY1*.*3* is enhanced as compared to wild type.

### EPS-independent twitching requires a low level of PilY1.1 and a high level of PilY1.3

Our transcriptomic results suggest that the restoration of motility by *ΔhsfA* and *ΔhsfB* in the absence of EPS is due to the combined down-regulation of *pilY1*.*1* and up-regulation of *pilY1*.*3*. Because null mutations in *pilY1*.*1* also restore motility in the absence of EPS, we wondered whether PilY1.3 protein levels were higher in both *ΔhsfA* and *ΔpilY1*.*1* as compared to wild type. Because it has been recently shown that PilY1.3 is located at the base and the tip of T4P [14], we attempted to measure the amounts of PilY1.3 present on pili from *ΔhsfA* and *ΔpilY1*.*1* cells.

For this, we constructed and placed at the endogenous locus a *pilY1*.*3-flag* fusion encoding a functional recombinant protein (S4A and S4B Fig, please also refer to the deletion mutants presented below). Then, to detect the presence of PilY1.3 on pili, we sheared fibers from the cell surface of a *pilY1*.*3-flag* strain. Pili were efficiently sheared from *pilY1*.*3-flag* cells as from wild type and PilY1.3-FLAG could be specifically detected by western blot with anti-FLAG antibodies (S4C Fig). The absence of PilC, a protein from the inner membrane, in the sheared pili fractions shows that these fractions were indeed not contaminated and that the detected PilY1.3-FLAG was exclusively extracellular (S4C Fig). Finally, we further confirmed the presence of Pil1.3-FLAG on pili tips by immunogold electron microscopy (S4D Fig).

**Fig 4:**
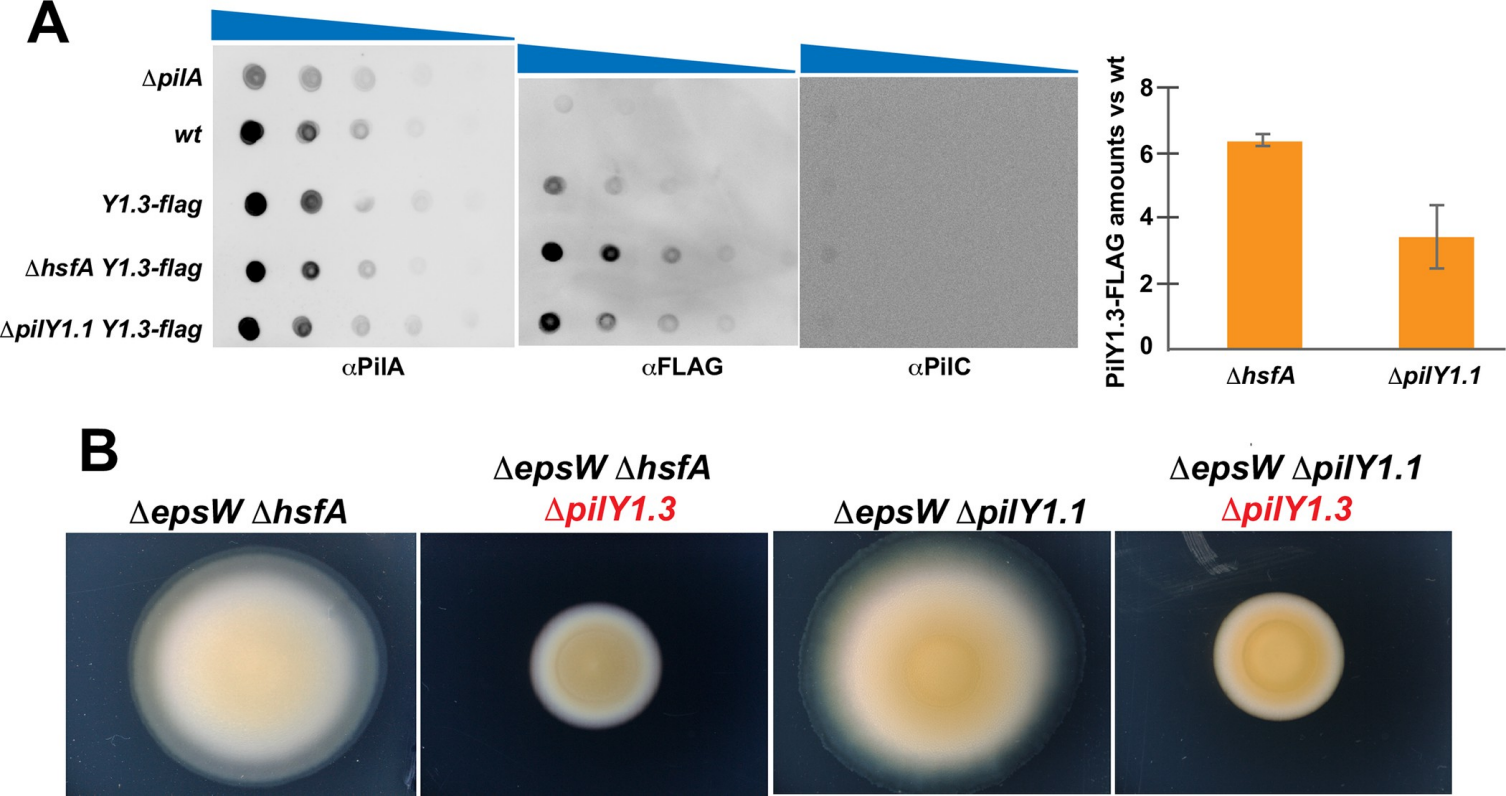
EPS-independent motility requires high levels of PilY1.3. (A) Sheared pili of the indicated strains were diluted as indicated and probed with αPilA (top panel), αFLAG (middle panel) and αPilC antibodies (bottom panel) for dot blots. The graphic shows, in a biological duplicate, the relative intensity of the FLAG signal in Δ*hsfA* and Δ*pilY1*.*1* vs wild type and normalized against the PilA signal. Error bars represent the standard deviation calculated on technical triplicates. Raw data are available in S4 Table. (B) Motility assays of EM810 (*ΔepsW ΔhsfA*), EM860 (*ΔepW ΔhsfA ΔpilY1*.*3*), EM813 (*ΔepsW ΔpilY1*.*1*) and EM864 (*ΔepW ΔpilY1*.*1 ΔpilY1*.*3*) on 0.5% agar at 72H.

With these tools to measure the PilY1.3 levels in *ΔhsfA* and *ΔpilY1*.*1*, we performed dot blot experiments on pilus fractions. Results show that normalized PilY1.3-FLAG amounts were 6.4 and 3.4 higher in *ΔhsfA* and *ΔpilY1*.*1*, respectively, than wild type (Fig 4A). This result supports the hypothesis that the emergence of twitching motility in the absence of EPS is due not only to the absence or down-regulation of *pilY1*.*1* but, likely, also to the over-representation of PilY1.3 on pili.

If restored twitching was exclusively due to PilY1.3, a deletion of *pilY1*.*3* from either *ΔepsW ΔpilY1*.*1* or *ΔepsW ΔhsfA* should lead to a complete loss of motility. As shown in Fig 4B, *ΔepsW ΔpilY1*.*1 ΔpilY1*.*3* and *ΔepsW ΔhsfA ΔpilY1*.*3* have no twitching motility (Fig 4B). These results indicate that PilY1.3 can promote twitching motility in the *ΔepsW ΔhsfA* and *ΔepsW ΔpilY1*.*1* mutants.

### PilY1.1 and PilY1.3 are involved in pilus assembly

Next, we aimed to investigate the roles of the PilY1.1 and PilY1.3 in EPS-dependent twitching motility. PiY1.1 and PilY1.3 are homologue and are both part of an operon also encoding four minor pilins (FimU, PilV, PilW, PilX) (S5 Fig). The co-expression and the functional association of PilY1.1 and PilW1 [14] explain the similar effects of their deletion on twitching (Fig 2A). The operon containing *pilY1*.*2* also contains homologues of the minor pilins (S5 Fig) [14].

First, we checked whether *ΔpilY1*.*2* and *ΔpilY1*.*3* could restore the motility defect of *ΔepsW* like *ΔpilY1*.*1*. *ΔepsW ΔpilY1*.*2* and *ΔepsW ΔpilY1*.*3* showed no net swarming similar to *ΔepsW* (S6 Fig), suggesting that *pilY1* genes do not have redundant functions in EPS-independent twitching.

**Fig 5:**
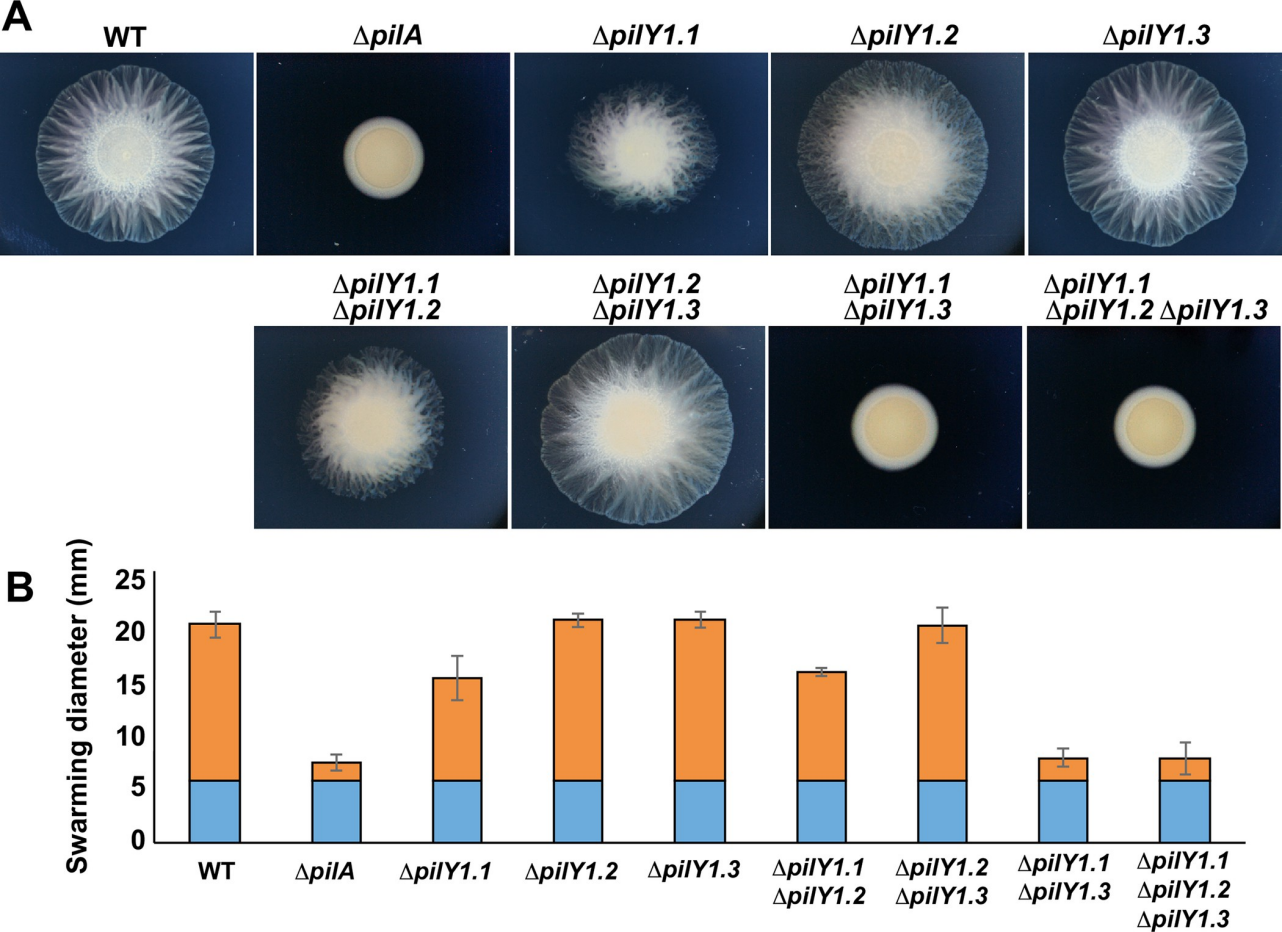
*pilY1*.*1* and *pilY1*.*3* have functions in twitching motility. (A) Motility phenotypes of DZ2 (wild type), TM389 (*ΔpilA*), EM808 (*ΔpilY1*.*1*), EM882 (*ΔpilY1*.*2*), EM856 (*ΔpilY1*.*3*), EM879 (*ΔpilY1*.*1 ΔpilY1*.*2*), EM880 (*ΔpilY1*.*2 ΔpilY1*.*3*), EM857 (*ΔpilY1*.*1 ΔpilY1*.*3*) and EM881 (*ΔpilY1*.*1 ΔpilY1*.*2 ΔpilY1*.*3*) on 0.5% agar and imaged at 48H. T4P-motile strains generate flares at the colony edge while non-motile strains form smooth-edged colonies. (B) Diameters of colonies of strains shown in (A). For each strain, the mean value of 3 biological replicates is plotted. Blue bar shows the diameters of the initial spots. Orange bar shows the swarm spreading. Raw data are available in S4 Table.

Then, we generated deletion mutants in each of *pilY1*.*1*, *pilY1*.*2* or *pilY1*.*3* genes and observed the resulting motility phenotypes. *ΔpilY1*.*2* and *ΔpilY1*.*3* showed no obvious motility defect, whereas *ΔpilY1*.*1* showed a reduced motility (Fig 5A and 5B), which is not due to a polar effect on *pilW1* (S7 Fig). This is different from what shown by Treuner-Lange and colleagues who observed no motility defects when mutating *pilY1*.*1* or its entire operon [14]. Such discrepancy might be due to the fact that Treuner-Lange et al. captured the colony expansion at 24h, a too early stage to resolve swarming differences between wild type and *ΔpilY1*.*1* (S8 Fig).

To check whether the mild or absent motility phenotypes of *pilY1* mutants were due to functional redundancy, we constructed all combinations of double mutants. *ΔpilY1*.*1 ΔpilY1*.*2* phenocopied *ΔpilY1*.*1*, and *ΔpilY1*.*2 ΔpilY1*.*3* showed no obvious phenotypes. However, *ΔpilY1*.*1 ΔpilY1*.*3* showed a complete loss of twitching motility, resembling a *ΔpilA* mutant (Fig 5A and 5B). Consistent with recent data [14], these results suggest that PilY1.1 and PilY1.3 have important functions in motility, whereas PilY1.2 is dispensable. This is agreement with our RNAseq data showing that *pilY1*.*2* expression in wild type was at the limit of detection being 2 log10 folds lower than that of *pilY1*.*1* and *pilY1*.*3*, with an average of 34,491 and 11,157 reads obtained for *pilY1*.*1* and *pilY1*.*3*, respectively, and only an average of 100 reads obtained for *pilY1*.*2*.

**Fig 6:**
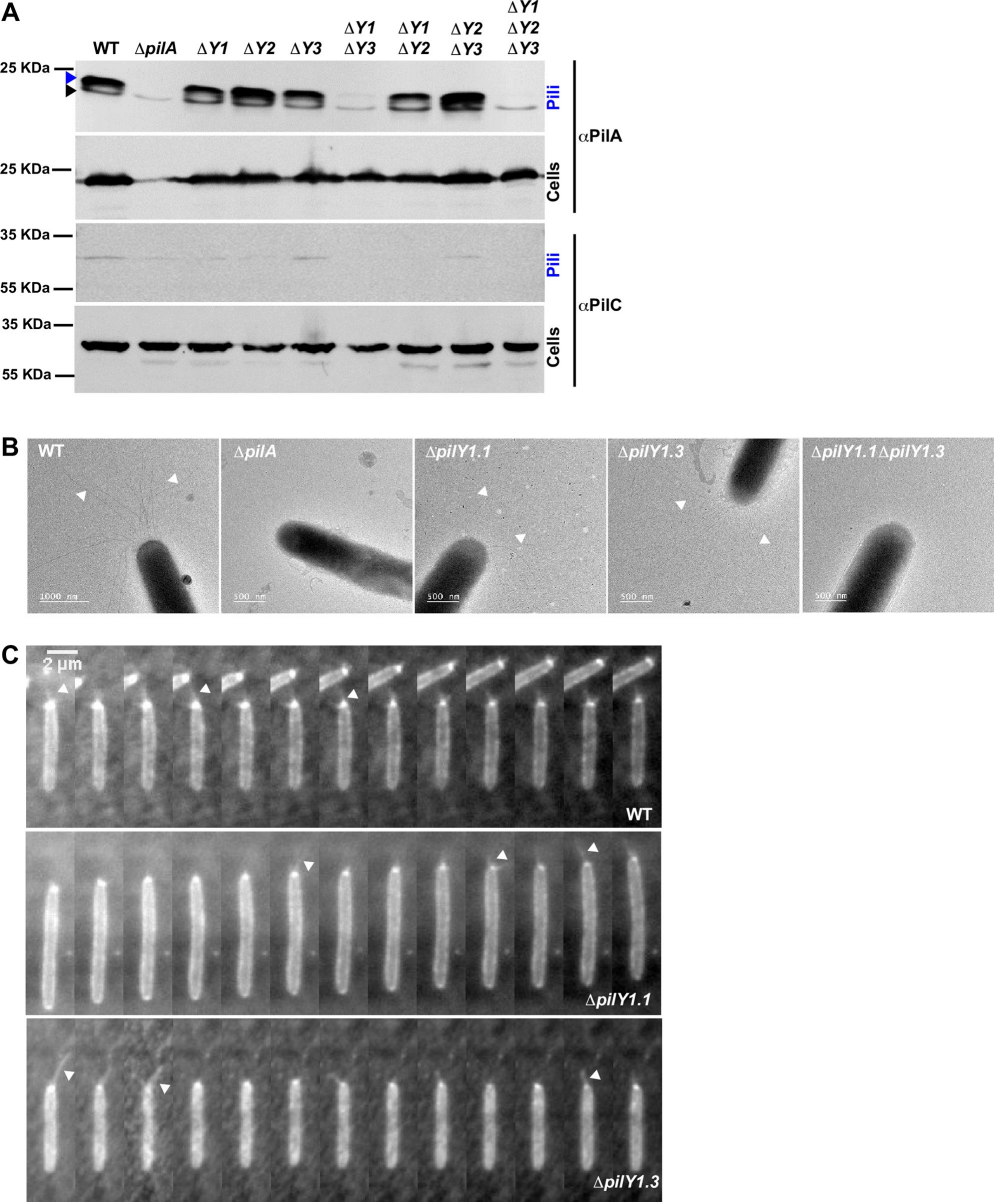
PilY1.1 and PilY1.3 are essential for T4P formation. (A) Western blot detection of PilA and PilC in sheared-off T4P and total cell lysates of DZ2 (wild type), TM389 (*ΔpilA*), EM808 (*ΔpilY1*.*1*), EM882 (*ΔpilY1*.*2*), EM856 (*ΔpilY1*.*3*), EM857 (*ΔpilY1*.*1 ΔpilY1*.*3*), EM879 (*ΔpilY1*.*1 ΔpilY1*.*2*), EM880 (*ΔpilY1*.*2 ΔpilY1*.*3*), and EM881 (*ΔpilY1*.*1 ΔpilY1*.*2 ΔpilY1*.*3*). Membranes were probed with αPilA antibodies (top rows) and αPilC antibodies (bottom rows). The blue arrowhead indicates the band corresponding to the PilA protein and the black arrowhead indicates a cross-reacting band. (B) TEM micrographs of cells from the indicated strains. Arrowheads indicate T4P on the cell surface. (C) 5s time-lapse series, obtained by TIRF microscopy, of labeled T4P pilin filaments and polar cluster enrichment of the indicated strains bearing att_mx8_::P_pilA_-pilA^D71C^.

To discriminate whether the concomitant absence of PilY1.1 and PilY1.3 led to the complete absence of pili or assembled but non-functional fibers, we sheared pili from the cell surface of different mutants and used the presence of PilA as read-out of pili assembly. Single *pilY1* deletion mutants as well as *ΔpilY1*.*1 ΔpilY1*.*2* and *ΔpilY1*.*2 ΔpilY1*.*3* double mutants all showed wild type levels of PilA both in the whole cell and surface fractions (Fig 6A). However, while PilA was still produced in *ΔpilY1*.*1 ΔpilY1*.*3*, it was not assembled on the cell surface of this mutant strain (Fig 6A). Because PilA monomers are stored in the inner membrane when not assembled in the fibers, we wanted to be sure that the sheared pili were not contaminated with inner membrane fractions. The absence of PilC in sheared pili shows that these fractions were indeed not contaminated and that the detected PilA was exclusively extracellular (Fig 6A). The direct visualization of pili by Electron Microscopy further confirmed the presence of pili on the surface of each strain but *ΔpilY1*.*1 ΔpilY1*.*3*, where no assembled fibers could be detected (Fig 6B).

Both Western Blot and Electron Microscopy showed that *ΔpilY1*.*1* assembles pili at wild-type levels (Fig 6A and 6B). Nevertheless, this strain shows a non-negligible reduction of motility on agar plates (Fig 5A). We thus decided to check the ability of pili of this strain to extend and retract by imaging T4P dynamics directly in single twitching cells as recently shown by Mercier and colleagues [26]. For this, we ectopically produced a PilA variant, PilA_D71C_, in each of our deletion strain, labeled the fibers with a cysteine-reactive maleimide fluorescent conjugate [6] and observed single cell twitching on glass slides by Total Internal Reflection Fluorescence (TIRF). Fig 6C and S7 and S8 Movies show the presence of fibers extending and retracting in *ΔpilY1*.*1* as in wild type. Taken together these results suggest that the motility defect observed in *ΔpilY1*.*1* is due neither to reduced amounts of pili nor to lack of the ability of T4P to extend and retract.

### A DUF4114 domain at the N terminus of PilY1.1 acts on twitching potentially upon Ca^2+^ binding

The absence of PilY1.1 leads to twitching motility defects at the colony level despite the ability of single mutant cells to assemble pili that extend and retract. We reasoned that the reduced swarming radius of *ΔpilY1*.*1* colonies might be due to an impaired ability of pili of this strain to bind EPS. To investigate this hypothesis, we searched for PilY1.1 protein features that would suggest a function in EPS-dependent twitching.

PilY1.1, PilY1.2 and PilY1.3 contain at the C terminus a PilY1 domain similar to PilY1 from *Pseudomonas aeruginosa* (3HX6) and at the N terminus a more variable domain: PilY1.3 contains a Von Willebrand factor A (VWA) domain [28], like PilY1 from *P*. *aeruginosa* [29], whereas PilY1.1 and PilY1.2 bear at their N terminus a domain of unknown function, DUF4114 (http://pfam.xfam.org/family/PF13448, [30]).

**Fig 7:**
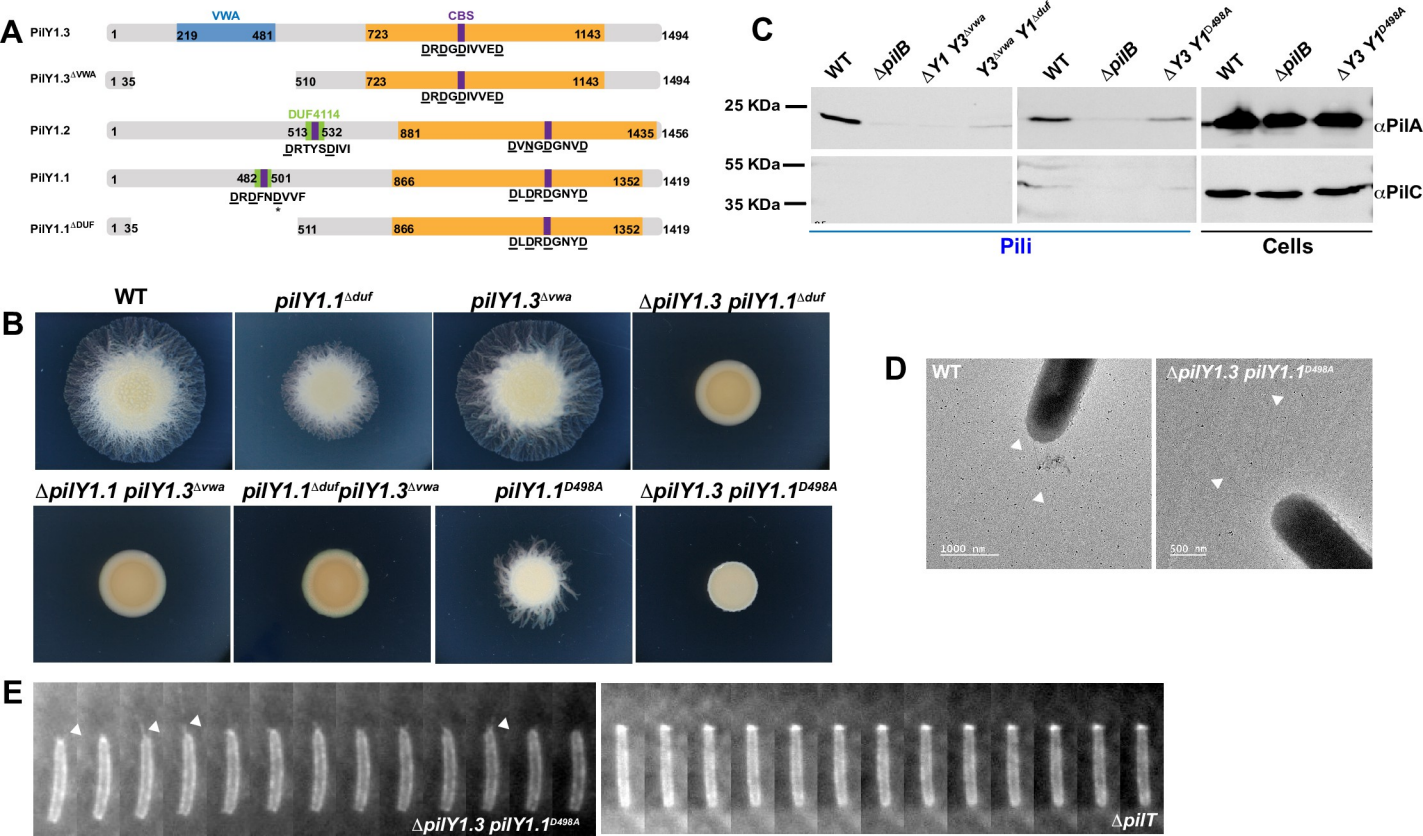
The C-terminal region of PilY1.1 and PilY1.3 is essential for T4P assembly. (A) Schematic representation of PilY1 proteins. Numbers at each side of the grey bars indicates the length of protein fragments. CBS (purple) stays for Calcium Binding Sites; VWA for Von Willbrand factor A (blue); DUF (green) stays for DUF4114; the conserved PilY1 domain is indicated in orange. The letters indicate the conserved protein sequences. The underlined amino acids indicate a consensus for a given motif. The star * corresponds to the conserved active D498 of PilY1.1. (B) Motility phenotypes of DZ2 (wild type), EM901 (*pilY1*.*1*^*Δduf*^), EM898 (*pilY1*.*3*^*Δvwa*^), EM902 (*ΔpilY1*.*3 pilY1*.*1*^*Δduf*^), EM897 (*ΔpilY1*.*1 pilY1*.*3*^*Δvwa*^), EM909 (*pilY1*.*1*^*Δduf*^ *pilY1*.*3*^*Δvwa*^), EM937 (*pilY1*.*1*^*D498A*^) and EM938 (*ΔpilY1*.*3 pilY1*.*1*^*D498A*^) on 0.5% agar and pictured at 48H. (C) Western blot detection of PilA and PilC in sheared-off T4P and total cell lysate of the indicated strains and using DZ2 (wild type) and EM747 (*ΔpilB*) as positive and negative controls, respectively. Membranes were probed with αPilA antibodies (top rows) and αPilC antibodies (bottom rows). (D) TEM micrographs of the indicated strains. Arrowheads indicate T4P on the cell surface. (E) 5s time-lapse series, obtained by TIRF microscopy, of labeled T4P pilin filaments and polar cluster enrichment of the indicated strains bearing att_mx8_::P_pilA_-pilA^D71C^.

Sequence alignment suggests the presence of a Ca^2+^ binding pocket in DUF4114, which is conserved in PilY1.1 but lacking an Asp in PilY1.2 (Fig 7A). DUF4114 is found in many bacterial sugar-binding proteins [31,32], including the mannose-binding lectins of the *Burkholderia* species (e.g. BclA) [33], *Ralstonia solanacearum* (RSIIL) [34] and *Pseudomonas aeruginosa* (PAIIL) [35], among others.

First, we deleted the N terminal domains from PilY1.1 and PilY1.3. Both *pilY1*.*1*^*Δduf*^ and *pilY1*.*3*^*Δvwa*^ phenocopied the respective null mutants in motility assays (please compare Fig 7B with Fig 5A). When these deletions were combined together or with a deletion of the corresponding homolog, none of the resulting *ΔpilY1 pilY1*.*3*^*Δvwa*^, *pilY1*.*1*^*Δduf*^ *ΔpilY1*.*3* and *pilY1*.*1*^*Δduf*^ *pilY1*.*3*^*Δvwa*^ double-mutant strains could support motility (Fig 7B). Moreover neither *pilY1*.*1*^*Δduf*^ *pilY1*.*3*^*Δvwa*^ nor *ΔpilY1 pilY1*.*3*^*Δvwa*^ produced pili like *ΔpilY1*.*1 ΔpilY1*.*3* (please compare Fig 7C with Fig 6A). This suggests that PilY1.1 and PilY1.3 are not functional in the absence of their respective N-terminal domains.

To further investigate a possible role of the PilY1.1 DUF4114 Ca^2+^ binding domain in EPS-dependent twitching, we deactivated it by generating a D to A amino acid substitution at position 498. This type of substitution usually abolishes the function of Ca^2+^ binding domains *in vivo* and *in vitro* [11, 36]. PilY1.1^D498A^ was expressed and had the expected size when fused to FLAG (*pilY1*.*1*^*D498A*^*-flag* was only used for western blot due to the absence of PilY1.1 antibodies, S4A Fig). Interestingly, while *pilY1*.*1*^*D498A*^ cells had the same motility defects as *ΔpilY1*.*1* at the colony level (Fig 7B), cells could assemble T4P even in the absence of PilY1.3 (Fig 7C–7E). Even if *ΔpilY1*.*3 pilY1*.*1*^*D498A*^ produced pili at lower amounts as compared to wild type (Fig 7C), these pili retained their ability to extend and retract (S9 Movie) unlike pili from *ΔpilT* cells [37] appearing as a single bright cluster at one cell pole in maleamide staining (Fig 7E and S10 Movie). These polar clusters, visible in all piliated strains, correspond to the MglA-dependent PilA membrane reservoir [26].

These results suggest that while the N terminal domain of PilY1.1 allows to produce net motility in the presence of EPS, potentially upon binding to Ca^2+^, the conserved C terminus is able to promote pilus assembly even in the absence of PilY1.3.

## Discussion

In this study, we used a genetic screen to find the protein targets of EpsW for the activation of EPS biosynthesis and export. Surprisingly, our genetic screen allowed the selections of suppressors whose T4P could mediate twitching in the absence of EPS. Because pili normally bind to EPS to promote motility in *M*. *xanthus*, we reasoned that pili from suppressor mutants allowed motility by binding a component of the extracellular matrix different than EPS. The selected point mutations lay in either of two operons: one encoding PilY1.1 and one encoding the HsfBA phosphorelay. All mutations led to null-mutant phenotypes. The similarity of *pilY1*.*1* and *hsfBA* suppression phenotypes is due to the fact that HsfBA up-regulates *pilY1*.*1* expression. Not only HsfBA up-regulates *pilY1*.*1* but it also down-regulates the expression of a *pilY1*.*1* homologue, *pilY1*.*3*. We show that the ability of pili to promote motility in the absence of EPS might be associated to the absence of *pilY1*.*1* or its down-regulation and to an accumulation of PilY1.3.

**Fig 8:**
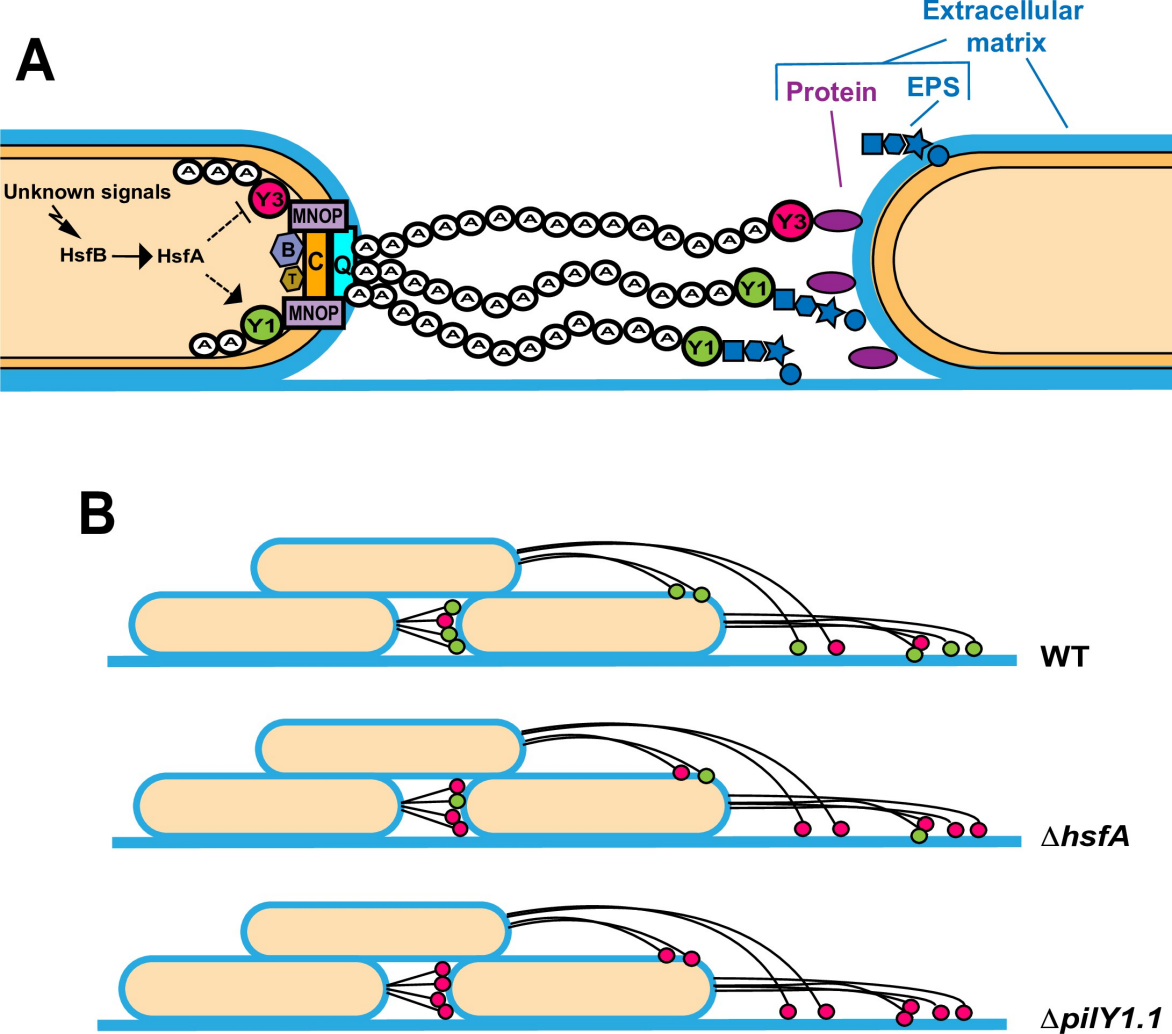
Proposed model on the regulation and role of PilY1 proteins. (A) Unknown environmental or metabolic signals induce the HsfBA two-component system that regulates positively the expression of PilY1.1 and negatively that of PilY1.3. PilY1.1 and PilY1.3 might be bi-functional proteins involved i) in priming T4P extension via their conserved C-terminal domains and putative interactions with cytoplasmic Pil components; ii) via their variable N-terminal domains and upon their translocation to the pilus tips, in adhering to components of the extracellular matrix deposited on the substrate or on the surface of neighboring cells. PilY1.1 might bind to EPS while PilY1.3 might bind to a different component of the matrix such as a protein or a nucleic acid. (B) In the absence (*ΔpilY1*.*1)* or low amounts (*ΔhsfA)* of PilY1.1, PilY1.3 could be preferentially assembled on T4P allowing motility thanks to the binding to substrates different than EPS.

In the light of our results and previous studies also showing the localization of PilY1.3 at the tip of the pilus [14], we speculate that the tip of the T4P fibers can be equipped of modular adhesion complexes that allow binding to different substrates according to the conditions and the bacterial cell needs (Fig 8A): as schematically shown in Fig 8B, in the absence or low amounts of PilY1.1, PilY1.3 could be preferentially assembled on T4P. This might be due to the fact that the pilus fiber, composed mainly of major pilin PilA, is a right-handed helix with a pitch consisting of ~3–4 PilA monomers and thought to be capped by four minor pilins (PilX, PilW, PilV and FimU) accomodating a molecule of PilY1 on the top [38]. Since there are two functional paralogs of PilY1 (PilY1.1 and PilY1.3), this suggests that there might be distinct pili, differing by the PilY paralog at their tip, e.g., when PilY1.1 is absent or in low amounts (in *ΔpilY1*.*1* or *ΔhsfA* mutants), pili will be capped by PilY1.3. Our results show that the restored motility observed in either *ΔepsW ΔpilY1*.*1* or *ΔepsW ΔhsfA* is indeed due to PilY1.3 activity.

PilY1.1 has two functions each exerted by a different protein domain: while the C terminus promotes the assembly of T4P, a function that is redundant with that of PilY1.3, the N terminus, containing a DUF4114 domain, could be responsible for promoting motility upon Ca^2+^ binding. DUF4114 domains are found in many bacterial sugar-binding proteins, including the mannose-binding lectins of the *Burkholderia* species (e.g. BclA) [33], *Ralstonia solanacearum* (RSIIL) [34] and *Pseudomonas aeruginosa* (PAIIL) [35]. We propose that this protein domain mediates the binding of pili to EPS to allow twitching motility in *M*. *xanthus*. Additional evidence that PilY1.1 might have adhesion properties comes from the fact that, while the inactivation of the putative DUF4114 Ca^2+^ binding motif leads to no motility at the colony level, active pili are assembled in single cells of this strain, even in the concomitant absence of PilY1.3. These pili, able to extend and retract even without binding to any substrate, leads to no net movement at the colony level. By analogy with other DUF4114-containing lectins, where sugar monomers directly participate in the coordination of Ca^2+^ ions, the binding of Ca^2+^ and sugars in PilY1.1 might be interdependent. For this reason, the inactivation of the Ca^2+^ binding site in the DUF4114 might lead to an impaired ability of PilY1.1 to bind its sugar substratum [33]. While the DUF4114 Ca^2+^ binding site might be important for sugar binding, the second Ca^2+^ binding site present in the PilY1 conserved C-terminal domain might be directly involved in priming T4P assembly [36].

PilY1.3 shows a modular organization similar to PilY1.1 but with a putative VWA domain at the N terminus replacing the DUF4114. This domain could bind to the second exopolysaccharide produced by *M*. *xanthus* cells, the BPS [17]. However we have arguments to believe that this is not the case: i) motility of a *ΔepsW ΔpilY1*.*1* is independent of BPS (S9 Fig); ii) VWA domains are often responsible for binding to proteins [28]. Interestingly, it has been recently shown that a minor pilin (PilB) from *Streptococcus sanguinis* bears a VWA domain at the C terminus involved in the binding to several human protein factors. This domain is, thus, responsible for the adhesion properties of *S*. *sanguinis* T4P [9]. It is possible that while PilY1.1 is required for binding to the exopolysaccharide fraction of the extracellular matrix, PilY1.3 binds to proteins. At least one protein, FibA, has been identified as part of the *M*. *xanthus* matrix [39]. Other proteins exposed on the cell surface might also bind PilY1.3.

Interestingly, while mutations in *pilY1*.*1* (and *hsfBA*) lead to improved motility in the absence of EPS, the motility of *ΔpilY1*.*1* is significantly impacted in a wild type background in the presence of EPS. We hypothesize that motility is less efficient in the presence of PilY1.3 alone because EPS has adhesion properties allowing cohesion between *M*. *xanthus* cells [17]. Such cohesion somehow counteracts the ability of cells to move in the presence of a less efficient twitching motility system. When the EPS counteraction is released, as in a *ΔepsW* background, *ΔpilY1*.*1* cells can move efficiently. Another possibility is that EPS prevents the access of PilY1.3 to its target(s).

While we could provide genetic evidences supporting a role of PilY1.1 in the binding to EPS, all our attempts to obtain functional full-length PilY1.1 or its N-terminal domain failed, preventing us from further testing such interaction *in vitro*. Also, while we could nicely observe PilY1.3 at the pilus tip, we could not obtain a functional FLAG fusion to localize PilY1.1 (S4B Fig). Nevertheless, the similarity between PilY1.1 and PilY1.3 supports the hypothesis that PilY1.1 is assembled on the pilus tip, like its homologue.

Finally our work provides an example of modularity of T4P where different PilY1 proteins can be assembled and their ratio transcriptionally regulated, presumably to adapt the pilus adhesion ability to the surrounding environment. Interestingly, it has been recently shown that *S*. *sanguinis* evolved a similar modularity by grafting a lectin and a von Willebrand factor A domain at the C terminus of either of two minor pilins, PilC and PilB, respectively [9]. This shows that distantly related bacterial species evolved T4P that by different strategies can adapt their adhesion ability to the environmental conditions.

## Methods

### Bacterial strains and growth conditions

The bacterial strains used in this study are listed in S2 Table. *Myxococcus xanthus* strains were grown at 32°C on Casitone-yeast extract (CYE) agar plates or in CYE liquid medium shaking at 160 rpm. 100ug ml-1 of kanamycin or 5 ug ml-1 of tetracycline was added into *M*. *xanthus* cultures when necessary. *Escherichia coli* cells were grown under standard laboratory conditions in LB media and supplemented with 25ug ml-1 of kanamycin and/or 100ug ml-1 ampicillin, where necessary.

### Plasmid and strain construction

The plasmids used in this work are listed in S3 Table. To create *M*. *xanthus* in-frame deletion strains, 900 bp upstream and downstream of the gene targeted for deletion were amplified by PCR and cloned into the pBJ114 [40] vector by the SLIC method [41]. To generate point mutation strains, a point mutation was designed in the primers. 900 bp upstream and downstream of this point mutation were amplified by PCR and the resulting fragments cloned into the pBJ114 vector by the SLIC method. To insert a FLAG tag to the C terminus of PilY1 proteins, the GATTACAAGGATGACGATGACAAG sequence (DYKDDDDK) was inserted in the primers to amplify the gene to tag. The resulting plasmids were then introduced into wild-type *M*. *xanthus* DZ2 via electroporation. Mutants and FLAG strains resulting from homologous recombination were obtained by selection on CYE agar plates containing first kanamycin and then galactose [40]. Point mutations were verified by Sanger sequencing (Eurofins GATC-Biotech). The FLAG fusion was verified by PCR using primer corresponding to GATTACAAGGATGACGATGACAAG sequence.

The plasmid to express *pilW1* under a vanillate inducible promoter was obtained by cloning the entire *pilW1* gene into plasmid pMR3690 [42].

*ΩcglB* [24] and *ΩpilA* [27] contains a *TnPhoA* transposon, with a Km^R^, in the *cglB* and *pilA* gene, respectively.

### Genetic screen

For selection of *ΔepsW* suppressor strains, a nonmotile *ΔepsWΩcglB* strain was grown in CYE medium until midexponential phase, and cells were concentrated to an OD_600_ of 10 in TPM buffer (10 mM Tris·HCl pH 7.6, 8 mM MgSO_4_, and 1 mM KH_2_PO_4_). Then cells were spotted (5 μL) on CYE 1.5% and 0.5% agar plates and incubated at 32°C for 2 weeks, until motile flares emerged from the colony. Flares of ΔepsW hsfA*, ΔepsW hsfB*, *ΔepsWpilY1*.1* and ΔepsWpilW1* strains were selected, their genomic DNA was extracted, and mutations were identified by whole-genome sequencing.

### Genome sequencing and identification of SNPs

Whole-genome sequencing was performed with the Illumina MiSeq System at the IMM Transcriptomic Platform. Sequencing samples were prepared using the Illumina Nextera XT DNA Library Preparation Kit according to the manufacturer’s instructions. Sequence reads were aligned with Unipro Ugene software [43] using the NCBI *M*. *xanthus* DK1622 genome (GenBank assembly accession no. GCA_000012685.1) as a reference. SNPs and nucleotide deletions/insertions were analyzed with Unipro Ugene software [43]. Genetic variations were confirmed by Sanger sequencing (Eurofins GATC-Biotech).

### Motility phenotypes

For motility phenotypic assays, exponentially growing cells in CYE medium at 32°C were adjusted to an OD_600_ of 10 in TPM buffer and spotted (5 μL) on CYE plates containing an agar concentration of 0.5%, incubated at 32°C, and photographed after 48 hours (for EPS-dependent motility) or 72 hours (for EPS-independent motility) with an Olympus SZ61 or Nikon Eclipse TE2000E microscope.

### Microscopy for colony swarming

For standard microscopy, exponentially growing cells grown in CYE media were washed, concentrated in TPM buffer and mounted on microscope slides covered with 1% CYE agarose pad. The cells were imaged on an automated and inverted epifluorescence microscope TE2000-E-PFS (Nikon), with a ×40/1.4 DLL objective and a camera orca flash 4 (Hamamatsu) at room temperature.

### Trypan Blue dye retention

Trypan Blue dye-retention analysis was performed as in [19]. Cells from overnight cultures were sedimented and resuspended in TPM buffer to OD_600_ 1.0, after which 900 μL of cell resuspension was transferred to a 1.5 mL microfuge tube; a cell-free blank was also prepared with an identical volume of TPM. To each tube, 100 μL of Trypan Blue stock solution (100 μg/mL) was added, followed by a brief 1 s pulse via vortex to mix the samples. All tubes were placed in a rack, covered with aluminum foil, and incubated at room temperature in the dark for 1 h. After this dye-binding period, samples were sedimented at high speed in a tabletop microfuge (16 000 × g, 5 min) to clear supernatants of intact cells. So as not to disrupt the pellet, only 900 μL of the dye-containing supernatant was aspirated and transferred to a disposable cuvette. The spectrophotometer was directly blanked at 585 nm (the absorption peak for Trypan Blue) using the cell-free “TPM + Trypan Blue” sample to calibrate the baseline absorbance corresponding to no retention of Trypan Blue dye by cells. Absorbance values at 585 nm (A585) were obtained for each clarified supernatant and normalized as a percentage of the WT A585 reading (i) as an internal control for each individual experiment, and (ii) to facilitate comparison of datasets across multiple biological replicates. Negative final values are due to trace amounts of cell debris detected at 585 nm in individual samples in which absolutely no binding of Trypan Blue occurred.

### Western and Dot Blot analysis

Total cell lysates were analyzed with SDS-PAGE (sodium dodecyl sulfate-polyacrylamide gel electrophoresis) and transferred to a nitrocellulose membrane. In the case of Dot Blot analyses, cell extracts were diluited and directly spotted on nitrocellulose membranes. After transfer, the membrane was blocked for 2 h at room temperature in 5% powdered milk. Proteins were detected using αPilA (1:10,000) [44], αPilC (1:5,000) [37] and FLAG antibody (1:1,000) (Anti-DDK rabbit polyclonal, OriGene Technologies Inc., Rockville, MD, USA) from rabbit in a mixture with TBST (Tris-buffered saline with Tween 20; OriGene Technologies Inc., Rockville, MD, USA) in combination with a secondary anti rabbit antibody (from goat) coupled to HRP (horseradish peroxidase) (1:5,000) (Thermo Fisher Scientific, MA, USA).

### Pili shearing assay

Pili were purified by shearing from cell surface pilin and prepared as described before [44] with slight modifications. The equivalent of 1ml of cells at a concentration of 1 OD_600_ was sedimented at 8,000 g for 5min and then resuspended in 1ml TPM buffer. The suspension was then vortexed at maximum speed with a table top vortexer for 30s to shear off pili and sedimented at 16,000 g for 1 min. The supernatant was then transferred to a fresh tube, centrifuged at 16,000 g for another 5 min and was then transferred to another fresh tube. Pili were precipitated by adding 100 mM MgCl_2_, incubating at 4°C overnight and sedimenting at 16,000 g for 15 min at 4°C.

### Protein purification

For protein purification with His_6_ tag, proteins of interest were cloned into pETPhos or pET28a+ vectors. The resulting plasmids were transformed into BL21 (DE3) cells. Over-expression was induced by adding 0.15mM IPTG to cell cultures and over-night incubation at 16°C. Cells were harvested and resuspended in lysis buffer (50 mM TrisHCl, pH 8.0; 300 mM NaCl; 100 μg/ml protease inhibitor; 30 U/mL Benzonase) and lysed at the French press. The cell lysates were centrifuged at 4°C for 20 min at 18000 g. Soluble tagged His_6_-proteins were purified on 0.5ml HisTrap Nickel columns (GE Healthcare) by washing and eluting with increasing concentrations of imidazole (10 mM, 75 mM, 200 mM and 500 mM imidazole) in 50 mM Tris-HCl pH 8.0 and 300 mM NaCl buffer. Pure fractions containing His_6_-protein were pooled and dialysed into a buffer containing 50 mM NaCl and 10 mM Tris pH 8.0.

### Electrophoretic mobility shift assay (EMSA)

DNA fragments used for EMSAs corresponding to 120-150bp upstream of the start codon of genes of interest were amplified by regular PCR with forward primers 5’ conjugated Alexa Fluor 488. For EMSAs, 10 nM PCR-amplification products were mixed with different concentrations of purified proteins in 1X binding buffer (10 mM Tris pH 8.0, 50mM KCl, 5% glycerol, 10 mM MgCl_2_) and then incubate at 4°C for 30min in dark before loading to native polyacrylamide gels (6.5%). Gel migration was performed in 1X TG buffer (25 mM Tris 192 mM Glycine pH 8.3) at 4°C for 40min under constant 250V. Gels were scanned with the Typhoon FLA 9500 imager (GE healthcare) using the following settings: excitation laser at 495 nm, emission filter 519 nm with LPB filter.

### RNA seq

For RNA extraction, cells were grown in CYE liquid medium at 32°C with shaking at 160rpm in the dark. Then, 1ml of cells at OD_600_ 1.0 were harvested by centrifuging at 8000rpm for 5min. Cells pellets were frozen by soaking in liquid nitrogen for 2min. RNA extraction was carried out using the Maxwell miRNA Tissue Kit (Promega) following the manufacturer’s instructions. Removal of rRNA and cDNA synthesis were carried out using Zymo-Seq RiboFree total RNA library Prep Kit (Zymo Research) following the manufacturer’s instructions. The cDNA from three biological triplicates of each sample was used for sequencing using the Illumina system (75bp paired-end reads) at the IMM Transcriptomic Platform. Sequence reads were pre-processed to remove low-quality bases and also cut the adaptors using Galaxy. Next, remaining reads were subsequently mapped to the *M*. *xanthus* DK1622 genome with the default parameters and using the pair-end strategy. Htseq-count were used to count the reads for each gene. SARtools and DEseq2 were used to analyze differential gene expression between wt and mutants. Differential expression was characterized by filtering with FDR < 0.01 and p < 0.001. RNAseq experiments were performed in three biological replicates.

### qRT-PCR

For RNA extraction, cells were grown in CYE liquid medium at 32°C with shaking at 160rpm in dark. Then, 1ml of cells at OD_600_ 1.0 were harvested by centrifuging at 8000rpm for 5min. Cells pellets were frozen by soaking in liquid nitrogen for 2min. RNA extraction was carried out using the Maxwell miRNA Tissue Kit (Promega) following the manufacturer’s instructions. To remove contaminated DNA, TURBO DNase (Invitrogen, RNase free) digestion step was performed. After that cDNA was synthesized using Zymo-Seq RiboFree total RNA library Prep Kit (Zymo Research) following the manufacturer’s instructions.

For cDNA synthesis, 1 μl of random hexamer primers (Invitrogen) and 500 ng of total RNA were denaturated at 95°C and chilled quickly on ice. A mix consisting of 4 μl of 5x buffer, 1 μl of RNase Inhibitor (Invitrogen), 1 μl of 5 mM dNTP and 1 μl of MMLV reverse transcriptase enzyme (200U/μl, Invitrogen) was added in a total volume of 20 μl, followed by 1 h of incubation at 45°C.

For all the reactions, qPCR conditions were identical and performed on a CFX96 Real-Time System (Bio-Rad). The reaction volume was 15 μL and the final concentration of each primer was 0.5 μM. The qPCR cycling parameters were 95°C for 2 min, followed by 45 cycles of 95°C for 5 s, 55°C for 60 s. A final melting curve from 65°C to 95°C was added to determine the specificity of the amplification. To determine the amplification kinetics of each product, the fluorescence derived from the incorporation of BRYT Green Dye into the double-stranded PCR products was measured at the end of each cycle using the GoTaq qPCR Master Mix 2X Kit (Promega). The data were analysed using Software Bio-Rad CFX manager 3.0 (BioRad), and the delta Ct method.

The 16s rRNA and constitutively expressed *frzE* genes were used as a reference for normalization. All measurements were carried out in triplicate and a biological duplicate was performed. Only reactions with over 80% efficiency were considered.

### Type IV Pilus labeling and observation

For type IV pili filament labeling, *M*. *xanthus* strains carrying the plasmid pSWU19-P_pilA_*pilA*^D71C^ [45] were grown in CYE medium until midexponential phase. Cells were injected in a preassembled Ibidi Sticky-Slide VI 0.4 microfluidic device sealed with a glass slide, coated with 0.015% carboxymethylcellulose [46]. After 30 min of incubation, Alexa Fluor 488 dye (Invitrogen) was added at 20 μg/mL in TPM buffer with 1 mM CaCl_2_ for 10 min in the dark, and cells were washed several times with TPM buffer with 1 mM CaCl_2_. Cells were imaged on a DeltaVision OMX SR Imaging system (GE Healthcare) in total internal reflection fluorescence (TIRF) mode with a 60× 1.49 NA TIRF objective and laser illumination (IMM Microscopy Platform).

Pictures and movies were prepared for publication using Fiji (https://fiji.sc/).

### Transmission electron microscopy (TEM)

To observe bacterial pili, 5 μl drops of exponentially growing *M*. *xanthus* cells in filtered CYE medium were placed directly on glow discharged carbon coated grids (EMS) for 3 minutes. The grids were then washed with ten drops of distilled water. Grids without further staining were dried on filter paper and the samples were analyzed using a Tecnai 200KV electron microscope (Thermo Fisher Scientific) and digital acquisitions were made with a numeric camera (16 megapixel, CMOS, Oneview, Gatan).

### Immunogold labeling of the Pili

To label PilY1 protein tagged with FLAG, exponentially growing *M*. *xanthus* cells in filtered CYE were gently spotted on carbon coated gold grids and allowed to settle for 20 min. The adhered cells fixed with 0.1% glutaraldehyde in PBS for 10 minutes. The grids subsequently were placed on drops: PBS–50 mM NH_4_Cl (5 min), PBS–1% bovine serum albumin (5 min), the primary antibody (αFLAG, anti-DDK rabbit polyclonal, OriGene Technologies Inc., Rockville, MD, USA) diluted 1/100 in PBS–1% BSA (1h), three washes in Aurion incubation buffer (2 min each), Prot G (Aurion, 10-nm-diameter gold particles) diluted 1/40 in incubation buffer (30 min), incubation buffer (3×3 min), PBS (3×5 min), glutaraldehyde 1% in PBS (10 min), dH_2_O (3×5 min). Grids without further staining were dried on filter paper and the samples were analyzed using a Tecnai 200KV electron microscope (Thermo Fisher Scientific), and digital acquisitions were made with a numeric camera (16 megapixel, CMOS, Oneview, Gatan).

## Supporting information

S1 Fig The HsfA regulon by RNAseq. Relative expression of the indicated genes in the Δ*hsfA* mutant measured as log2-fold changes by RNA-Seq. The plot shows genes that, from the RNAseq results are up- or down-regulated of at least 3 log_2_-fold changes. Results are mean values from three biological replicates. The green and red colors indicate up- and down-regulation, respectively, as compared to wild type.
(TIF)

S2 Fig His6-HsfA can be heterologously expressed and purified. SDS page showing samples from the different steps of His_6_-HsfA purification. The mM values indicate the imidazole concentrations at which the different elution steps were performed. MW stays for Molecular Weight. The eluted protein migrated at the expected size (55.7 kDa).
(TIF)

S3 Fig Role of specific and non-specific DNA in EMSA assays. Representative Electrophoretic mobility shift assays (EMSA) on 6.5% polyacrylamide. The indicated concentrations of purified His_6_-HsfA were incubated with the indicated DNA fragments. (A) Specific unlabeled P_*pilY1*.*1*_ and P_*pilY1*.*3*_ fragments were added at the indicated concentrations. (B) Poly-deoxy-inosinic-deoxy-cytidylic acid (Poly dI-dC) was added at the indicated concentrations.
(TIF)

S4 Fig PilY1.3-FLAG is stably expressed and functional. (A) Western blot detection of FLAG-PilY1 fusions in total cell lysate of DZ2 (wild type), EM871 (*pilY1*.*3-FLAG*), EM876 (*pilY1*.*1-FLAG*), EM883 (*pilY1*.*2-FLAG*) and EM941 (*pilY1*.*1* ^*D498A*^ *-FLAG*). (B) Motility phenotypic assays of DZ2 (wild type), EM876 (*pilY1*.*1-FLAG*), EM915 (*ΔpilY1*.*3 pilY1*.*1-FLAG*), EM871 (*pilY1*.*3-FLAG*) and EM905 (*ΔpilY1*.*1 pilY1*.*13-FLAG*) on 0.5% agar at 48H. (C) Western blot detection of PilA, Pil1.3-FLAG and PilC in sheared-off T4P and total cell lysate of the indicated strains. Membranes were probed with αPilA, αFLAG and αPilC antibodies. The blue arrow indicates the band corresponding to the PilA protein and the black arrow indicates a cross-reacting band (please also refer to Fig 6A). (D) Immunogold labeling experiment on *pilY1*.*3-flag* cells. Cells were probed with αFLAG primary antibodies followed by secondary antibodies conjugated to gold particles. Samples were imaged by TEM. Arrowheads indicate gold particles associated with T4P tips. The experiment was repeated 3 times.
(TIF)

S5 Fig Schematic representation of *M. xanthus* gene clusters encoding PilY1 and minor pilins homologs. *pilY1* and *pilW* genes are shown in blue.
(TIF)

S6 Fig *pilY1* genes do not have redundant functions in EPS-independent twitching. Motility phenotypes of EM605 (*ΔepsW*), EM813 (*ΔepsW ΔpilY1*.*1*), EM861 (*ΔepsW ΔpilY1*.*2*) and EM884 (*ΔepsW ΔpilY1*.*3*) on 0.5% agar and imaged at 72H.
(TIF)

S7 Fig A Pvan-*pilW1* gene can restore the motility defects of *ΔpilW1* but not that of *ΔpilY1.1*. Motility phenotypes of DZ2 (wild type), EM831 (*ΔpilW1*), EM977 (*ΔpilW1 Pvan-pilW1*), EM808 (*ΔpilY1*.*1*) and EM978 (*ΔpilY1*.*1 Pvan-pilW1*) on 0.5% agar imaged at 48H.
(TIFF)

S8 Fig The *pilY1.1* motility defects can be resolved upon a 48h incubation. Motility phenotypes of DZ2 (wild type) and EM808 (*ΔpilY1*.*1*) on 0.5% agar imaged at 24H and 48H.
(TIF)

S9 Fig BPS is not involved in EPS-independent twitching motility. Motility phenotypes of EM749 (*ΔepsW ΩcglB*), EM 872 (*ΔepsW ΔpilY1*.*1 ΩcglB*) and EM975 (*ΔepsW ΩcglB ΔpilY1*.*1 ΩwzaB*) on 0.5% agar at 48H.
(TIF)

S1 Movie 30 sec time-lapse series of a *ΔepsW ΩcglB* mini colony. (AVI)

S2 Movie 30 sec time-lapse series of a *ΔpilA ΩcglB* mini colony. (AVI)

S3 Movie 30 sec time-lapse series of a ΔepsW ΩcglB hsfB* *(suppC4)* mini colony. (AVI)

S4 Movie 30 sec time-lapse series of a ΔepsW ΩcglB hsfA* mini colony. (AVI)

S5 Movie 30 sec time-lapse series of a ΔepsW ΩcglB pilY1.1* mini colony. (AVI)

S6 Movie 30 sec time-lapse series of a ΔepsW ΩcglB pilW1* mini colony. (AVI)

S7 Movie 5s time-lapse series, obtained by TIRF microscopy, of labeled T4P pilin filaments and polar cluster enrichment of *M. xanthus* wild type cells bearing *attmx8::PpilA-pilAD71C*. (AVI)

S8 Movie 5s time-lapse series, obtained by TIRF microscopy, of labeled T4P pilin filaments and polar cluster enrichment of *ΔpilY1.1* bearing *attmx8::PpilA-pilAD71C*. (AVI)

S9 Movie 5s time-lapse series, obtained by TIRF microscopy, of labeled T4P pilin filaments and polar cluster enrichment of *ΔpilY1.3 pilY1.1^D498A^* bearing *attmx8::PpilA-pilAD71C*. (AVI)

S10 Movie 5s time-lapse series, obtained by TIRF microscopy, of labeled T4P pilin filaments and polar cluster enrichment of *ΔpilT* bearing *attmx8::PpilA-pilAD71C*. (AVI)

S1 Table RNAseq results including the relative expression of the indicated genes in the Δ*hsfA* mutant measured as log2-fold changes as compared to wild type. Results are mean values from three biological replicates.
(XLSX)

S2 Table Strains used in this work. (PDF)

S3 Table Plasmids used in this work. (PDF)

S4 Table Raw data for plots on Figs 2B, 3D, 4A and 5B. (XLSX)
