# Supplementary figures and images for: The differential expression of PilY1 proteins by the HsfBA phosphorelay allows twitching motility in the absence of exopolysaccharides
Source: PLoS Genet. 2022 Apr 29;18(4):e1010188. doi: 10.1371/journal.pgen.1010188 (PMC9109919; doi:10.1371/journal.pgen.1010188)

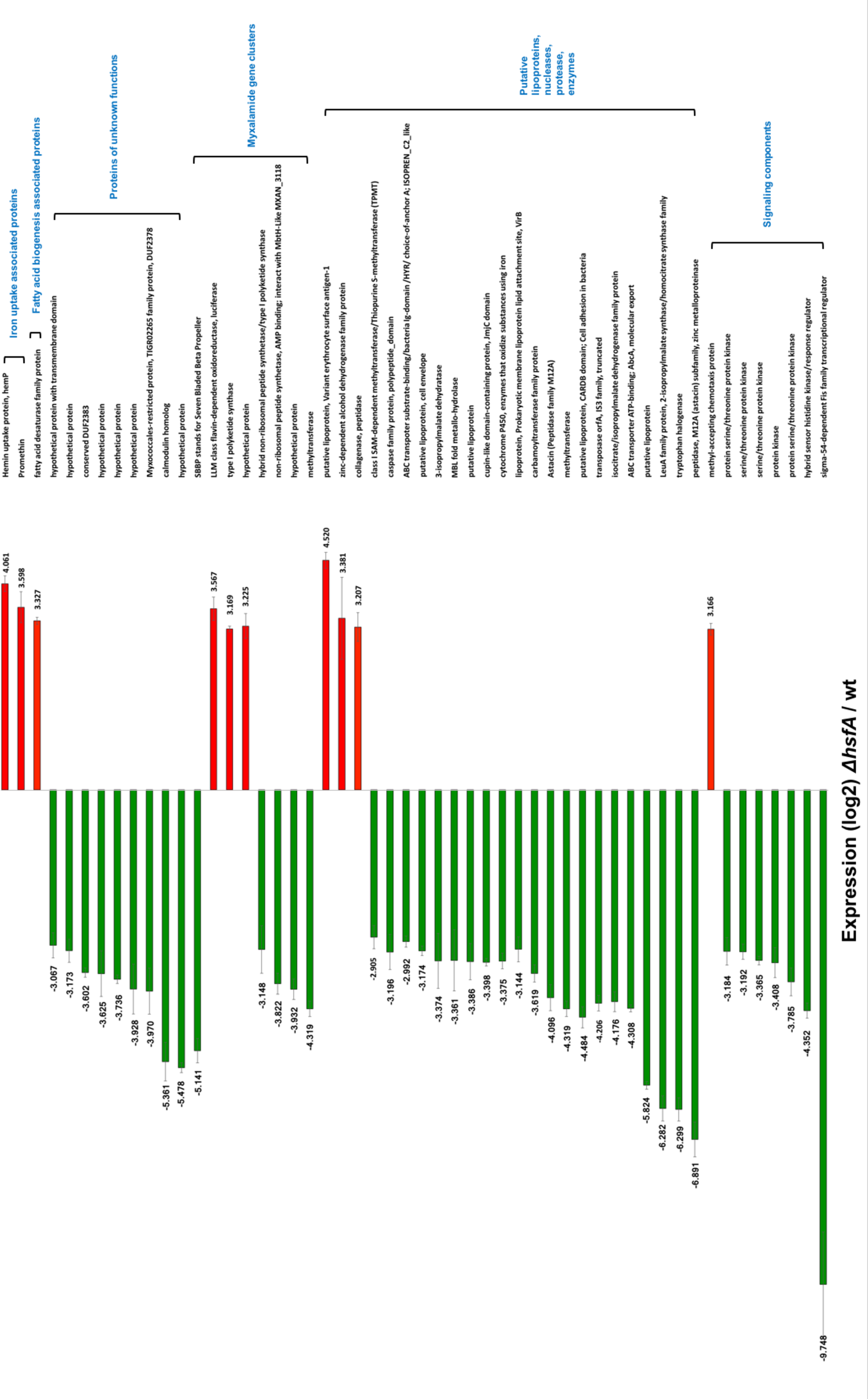

Supplement: S1 Fig — Relative expression of the indicated genes in the ΔhsfA mutant measured as log2-fold changes by RNA-Seq. The plot shows genes that, from the RNAseq results are up- or down-regulated of at least 3 log2-fold changes. Results are mean values from three biological replicates. The green and red colors indicate up- and down-regulation, respectively, as compared to wild type. (TIF) [file pgen.1010188.s001.tif]

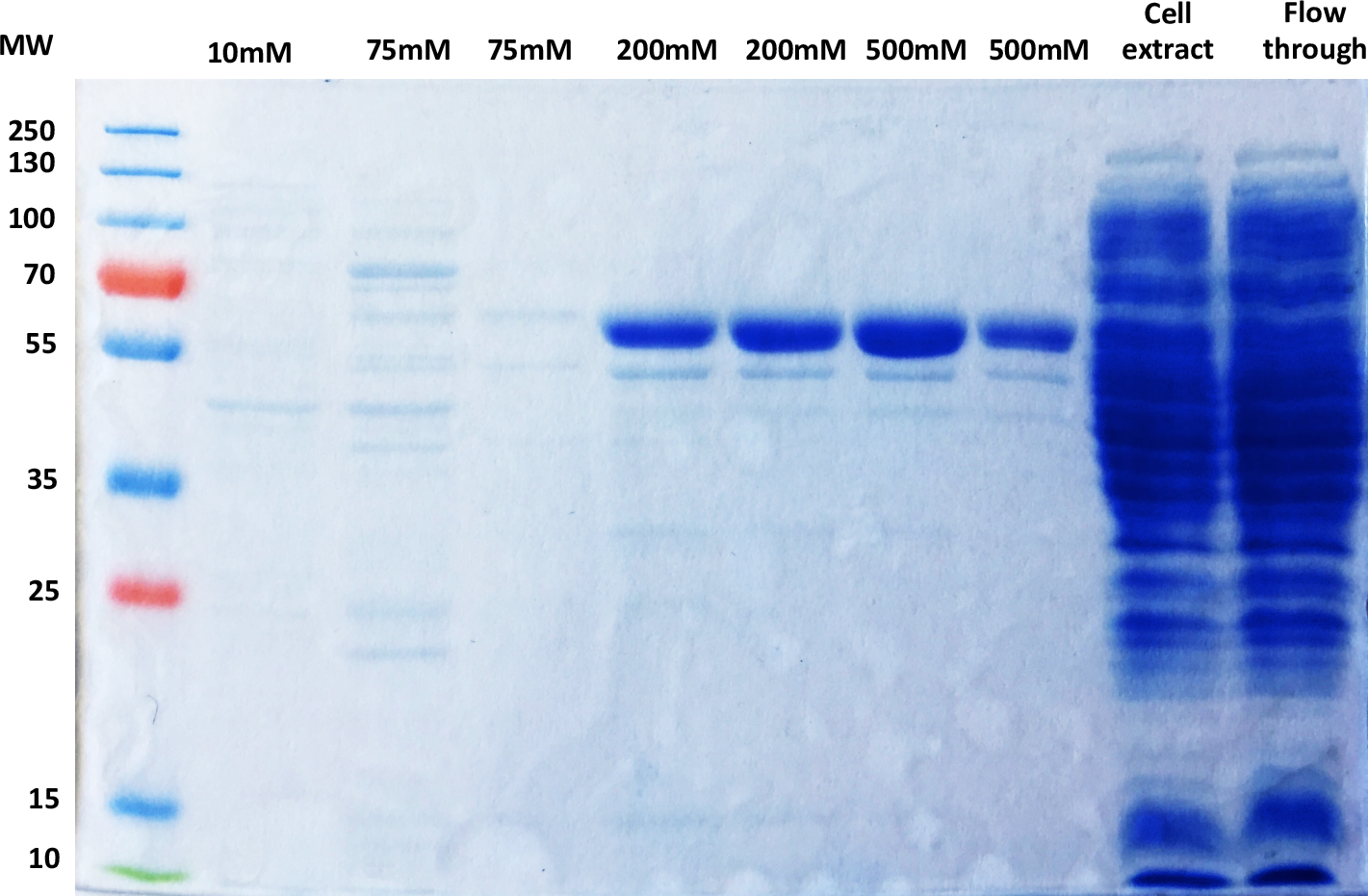

Supplement: S2 Fig — SDS page showing samples from the different steps of His6-HsfA purification. The mM values indicate the imidazole concentrations at which the different elution steps were performed. MW stays for Molecular Weight. The eluted protein migrated at the expected size (55.7 kDa). (TIF) [file pgen.1010188.s002.tif]

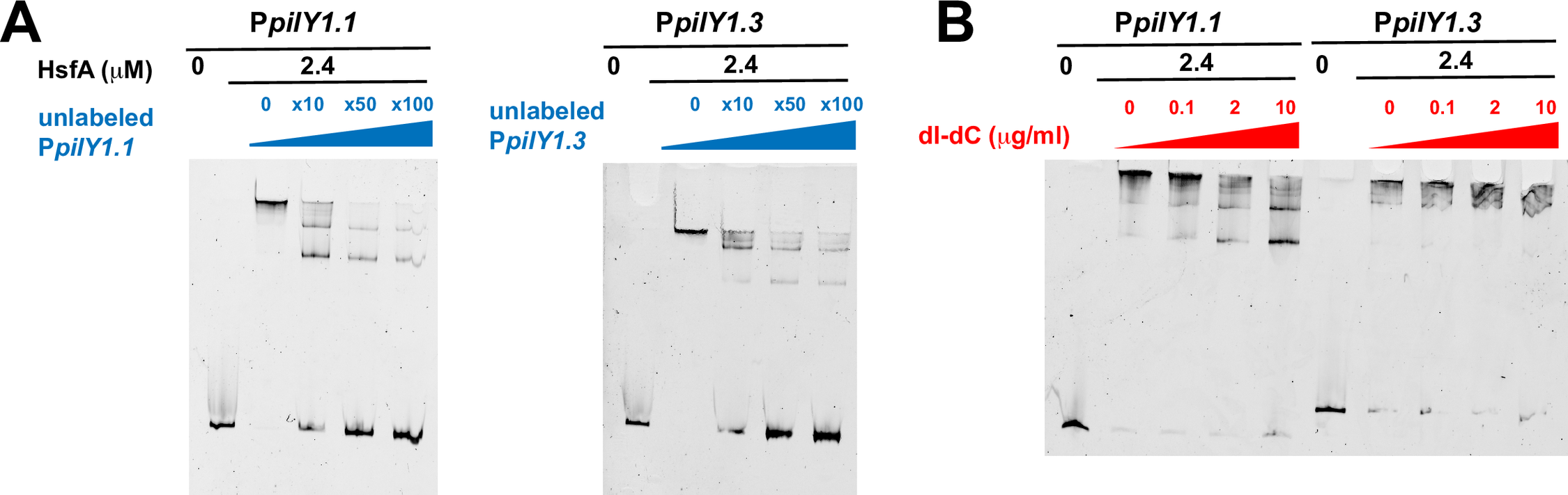

Supplement: S3 Fig — Representative Electrophoretic mobility shift assays (EMSA) on 6.5% polyacrylamide. The indicated concentrations of purified His6-HsfA were incubated with the indicated DNA fragments. (A) Specific unlabeled PpilY1.1 and PpilY1.3 fragments were added at the indicated concentrations. (B) Poly-deoxy-inosinic-deoxy-cytidylic acid (Poly dI-dC) was added at the indicated concentrations. (TIF) [file pgen.1010188.s003.tif]

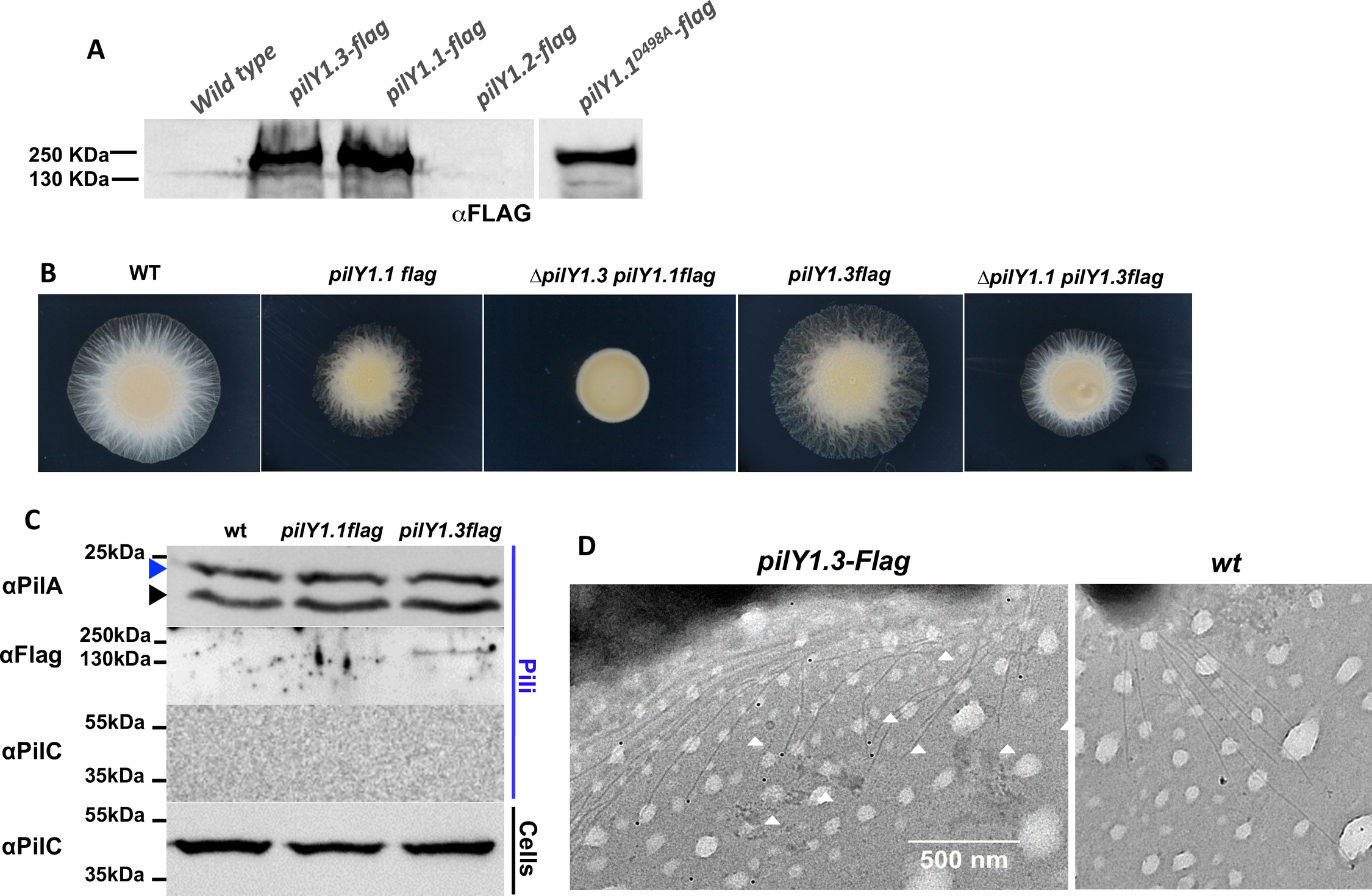

Supplement: S4 Fig — (A) Western blot detection of FLAG-PilY1 fusions in total cell lysate of DZ2 (wild type), EM871 (pilY1.3-FLAG), EM876 (pilY1.1-FLAG), EM883 (pilY1.2-FLAG) and EM941 (pilY1.1 D498A -FLAG). (B) Motility phenotypic assays of DZ2 (wild type), EM876 (pilY1.1-FLAG), EM915 (ΔpilY1.3 pilY1.1-FLAG), EM871 (pilY1.3-FLAG) and EM905 (ΔpilY1.1 pilY1.13-FLAG) on 0.5% agar at 48H. (C) Western blot detection of PilA, Pil1.3-FLAG and PilC in sheared-off T4P and total cell lysate of the indicated strains. Membranes were probed with αPilA, αFLAG and αPilC antibodies. The blue arrow indicates the band corresponding to the PilA protein and the black arrow indicates a cross-reacting band (please also refer to Fig 6A). (D) Immunogold labeling experiment on pilY1.3-flag cells. Cells were probed with αFLAG primary antibodies followed by secondary antibodies conjugated to gold particles. Samples were imaged by TEM. Arrowheads indicate gold particles associated with T4P tips. The experiment was repeated 3 times. (TIF) [file pgen.1010188.s004.tif]

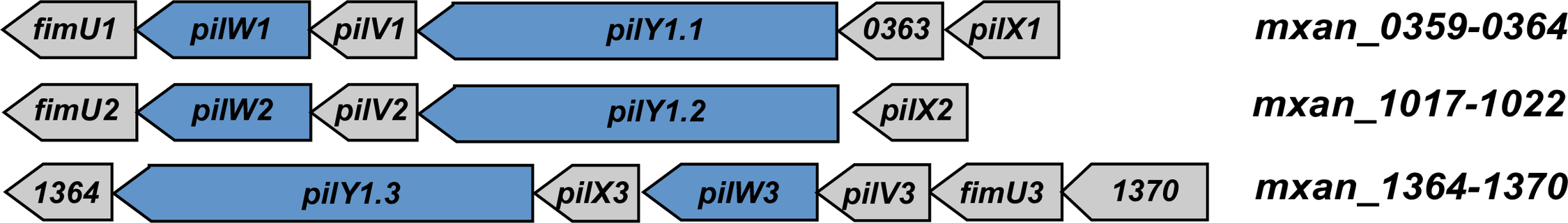

Supplement: S5 Fig — pilY1 and pilW genes are shown in blue. (TIF) [file pgen.1010188.s005.tif]

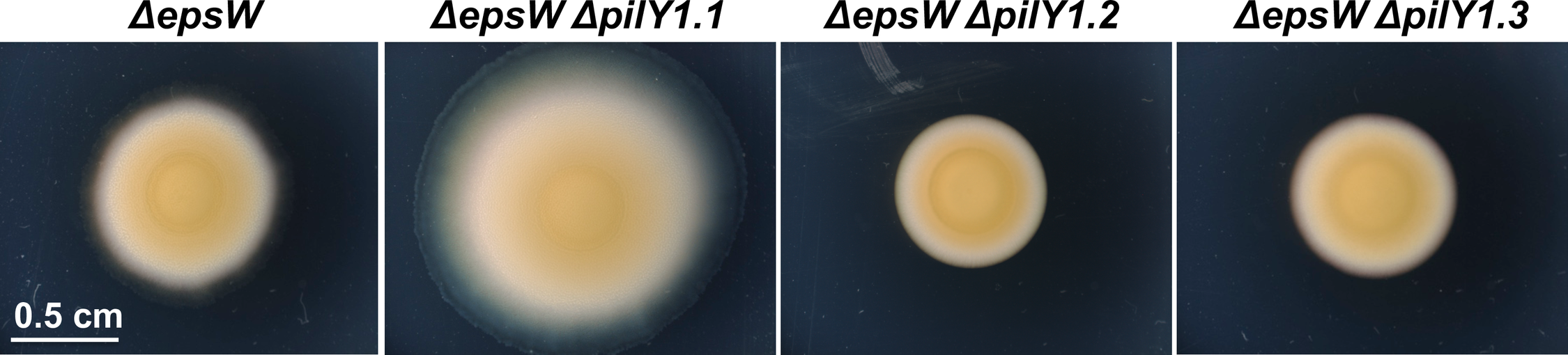

Supplement: S6 Fig — Motility phenotypes of EM605 (ΔepsW), EM813 (ΔepsW ΔpilY1.1), EM861 (ΔepsW ΔpilY1.2) and EM884 (ΔepsW ΔpilY1.3) on 0.5% agar and imaged at 72H. (TIF) [file pgen.1010188.s006.tif]

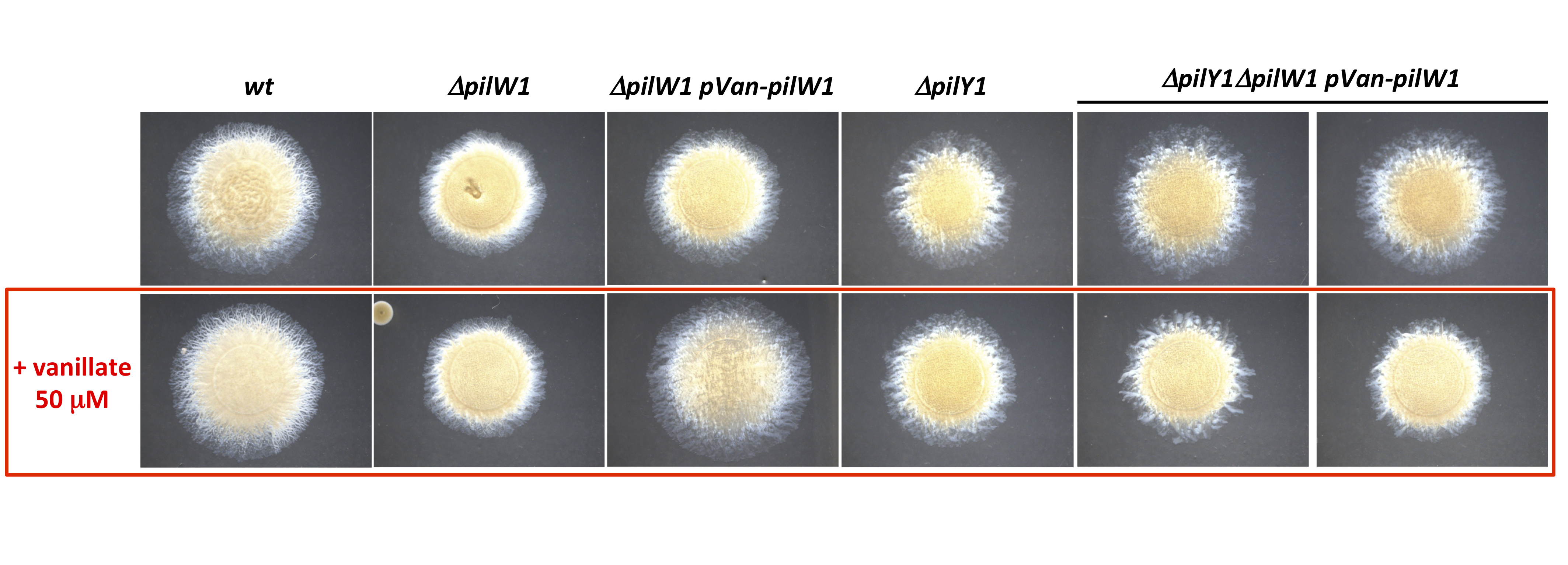

Supplement: S7 Fig — Motility phenotypes of DZ2 (wild type), EM831 (ΔpilW1), EM977 (ΔpilW1 Pvan-pilW1), EM808 (ΔpilY1.1) and EM978 (ΔpilY1.1 Pvan-pilW1) on 0.5% agar imaged at 48H. (TIFF) [file pgen.1010188.s007.tiff]

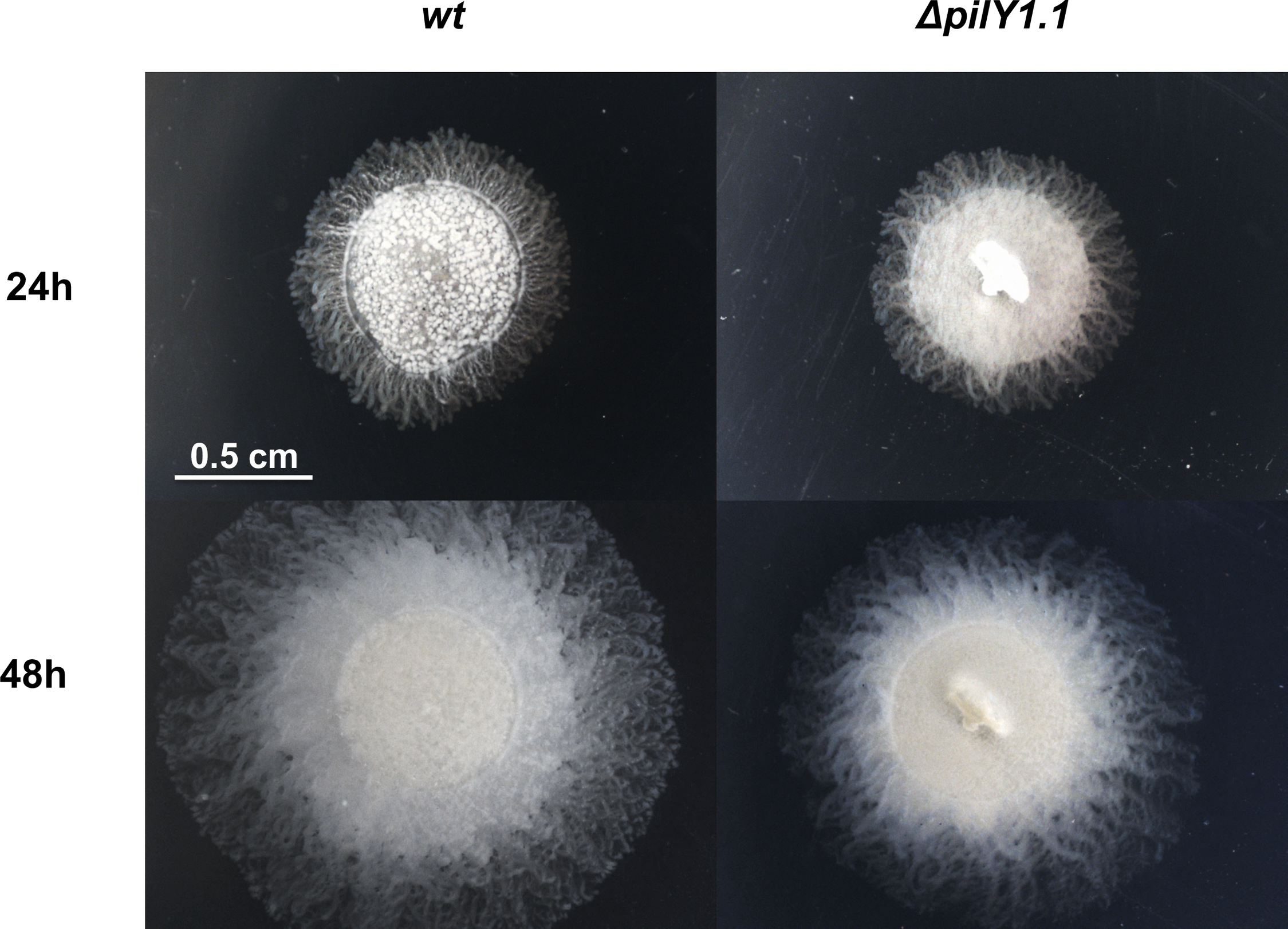

Supplement: S8 Fig — Motility phenotypes of DZ2 (wild type) and EM808 (ΔpilY1.1) on 0.5% agar imaged at 24H and 48H. (TIF) [file pgen.1010188.s008.tif]

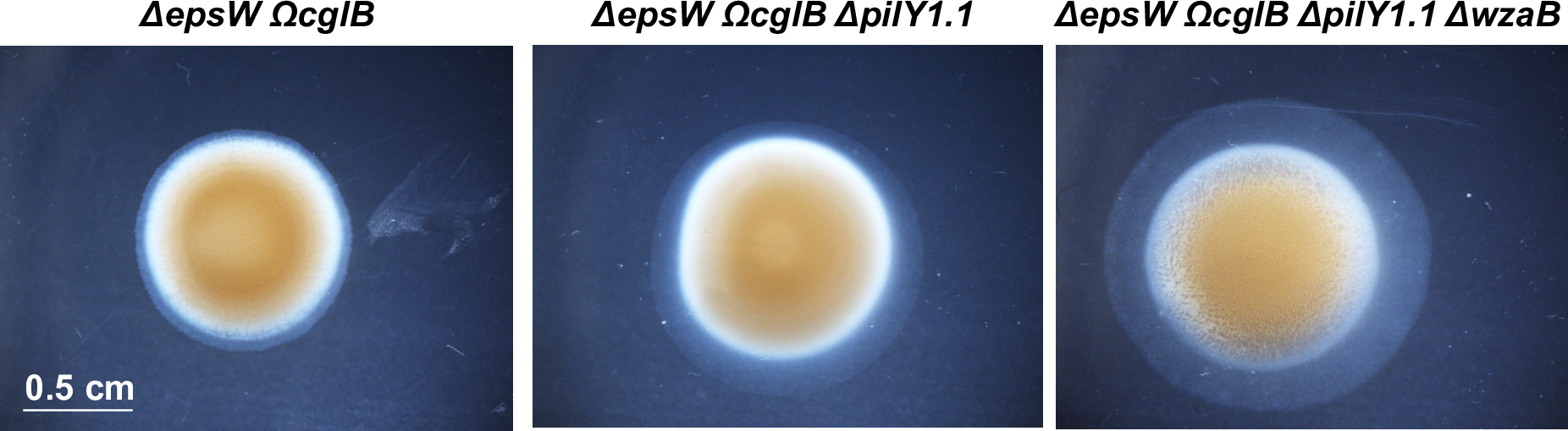

Supplement: S9 Fig — Motility phenotypes of EM749 (ΔepsW ΩcglB), EM 872 (ΔepsW ΔpilY1.1 ΩcglB) and EM975 (ΔepsW ΩcglB ΔpilY1.1 ΩwzaB) on 0.5% agar at 48H. (TIF) [file pgen.1010188.s009.tif]
