# Supplementary material for: The differential expression of PilY1 proteins by the HsfBA phosphorelay allows twitching motility in the absence of exopolysaccharides
Source: PLoS Genet. 2022 Apr 29;18(4):e1010188. doi: 10.1371/journal.pgen.1010188 (PMC9109919; doi:10.1371/journal.pgen.1010188)
Supplement: S2 Table — (PDF) [file pgen.1010188.s021.pdf]

Supplementary Table S2

**Strains**	**Genotype**	**Reference or Source**
TM108	*Myxococcus xanthus DZ2*, Wild type	1
EM650	*ΔepsW*	2
TM770	*ΩcglB Km*	Laboratory collection
TM389	*ΔpilA*	3
TM294	*ΩpilA Tc*	3
EM747	*ΔpilB*	4
EM589	*ΔpilT*	This study
EM749	*ΔepsW ΩcglB*	This study
EM785	*ΔepsW ΩcglB sup4*	This study
EM788	*ΔepsW ΩcglB sup7*	This study
EM789	*ΔepsW ΩcglB sup8*	This study
EM790	*ΔepsW ΩcglB sup9*	This study
EM791	*ΔepsW ΩcglB sup10*	This study
EM792	*ΔepsW ΩcglB sup11*	This study
EM793	*ΔepsW ΩcglB sup12*	This study
EM803	*ΔepsW hsfB*C4 (from sup4)*	This study
EM809	*ΔepsW hsfB*C7 (from sup7)*	This study
EM818	*ΔepsW hsfA* (from sup9)*	This study
EM804	*ΔepsW pilW1* (from sup8)*	This study
EM805	*ΔepsW pilY1.1* (from sup10/11/12)*	This study
EM829	*ΔepsW hsfB*1 ΩcglB*	This study
EM830	*ΔepsW hsfB*2 ΩcglB*	This study
EM874	*ΔepsW hsfA* ΩcglB*	This study
EM840	*ΔepsW pilW1* ΩcglB*	This study
EM832	*ΔepsW pilY1.1* ΩcglB*	This study
EM823	*ΔepsW hsfB*1 ΩpilA*	This study
EM824	*ΔepsW hsfB*2 ΩpilA*	This study
EM943	*ΔepsW hsfA* ΩpilA*	This study
EM825	*ΔepsW pilW1* ΩpilA*	This study
EM826	*ΔepsW pilY1.1* ΩpilA*	This study
EM811	*ΔepsW ΔhsfB*	This study
EM810	*ΔepsW ΔhsfA*	This study
EM845	*ΔepsW ΔpilW1*	This study
EM813	*ΔepsW ΔpilY1.1*	This study
EM833	*ΔepsW ΔhsfA ΩcglB*	This study

EM834	*ΔepsW ΔhsfB ΩcglB*	This study
EM872	*ΔepsW ΔpilY1.1 ΩcglB*	This study
EM873	*ΔepsW ΔpilW1 ΩcglB*	This study
EM827	*ΔepsW ΔhsfA ΩpilA*	This study
EM828	*ΔepsW ΔhsfB ΩpilA*	This study
EM942	*ΔepsW ΔpilY1.1 ΩpilA*	This study
EM944	*ΔepsW ΔpilW1 ΩpilA*	This study
EM806	*ΔhsfA*	This study
EM807	*ΔhsfB*	This study
EM817	*hsfA* (from sup9)*	This study
EM799	*hsfB*C4 (from sup4)*	This study
EM816	*hsfB*C7 (from sup7)*	This study
EM831	*ΔpilW1*	This study
EM808	*ΔpilY1.1*	This study
EM882	*ΔpilY1.2*	This study
EM856	*ΔpilY1.3*	This study
EM879	*ΔpilY1.1 ΔpilY1.2*	This study
EM857	*ΔpilY1.1 ΔpilY1.3*	This study
EM880	*ΔpilY1.2 ΔpilY1.3*	This study
EM881	*ΔpilY1.1 ΔpilY1.2 ΔpilY1.3*	This study
EM876	*pilY1.1-FLAG*	This study
EM883	*pilY1.2-FLAG*	This study
EM871	*pilY1.3-FLAG*	This study
EM941	*pilY1.1*^{D498A}*-FLAG*	This study
EM915	*ΔpilY1.3 pilY1.1-FLAG*	This study
EM905	*ΔpilY1.1 pilY1.13-FLAG*	This study
EM916	*DZ2* $P_{pilA}PilA^{D71C}$	This study
EM918	*ΔpilY1.1* $P_{pilA}PilA^{D71C}$	This study
EM919	*ΔpilY1.3* $P_{pilA}PilA^{D71C}$	This study
EM927	*ΔpilT* $P_{pilA}PilA^{D71C}$	This study
EM901	*pilY1.1*$^{\Delta duf}$	This study
EM898	*pilY1.3*$^{\Delta vwa}$	This study
EM902	*ΔpilY1.3 pilY1.1*$^{\Delta duf}$	This study
EM897	*ΔpilY1.1 pilY1.3*$^{\Delta vwa}$	This study
EM909	*pilY1.1*$^{\Delta duf}$ *pilY1.3*$^{\Delta vwa}$	This study
EM937	*pilY1.1*^{D498A}	This study
EM938	*ΔpilY1.3 pilY1.1*^{D498A}	This study

EM945	*ΔpilY1.3 pilY1.1*^{D498A} P_{pilA}*pilA*^{D71C}	This study
EM864	*ΔepW ΔpilY1.1 ΔpilY1.3*	This study
EM860	*ΔepW ΔhsfA ΔpilY1.3*	This study
EM861	*ΔepW ΔpilY1.3*	This study
EM884	*ΔepW ΔpilY1.2*	This study
EM975	*ΔepsW ΩcglB ΔpilY1.1 ΩwzaB*	This study
EM977	*ΔpilW1 pVan-pilW1* (pEM668)	This study
EM978	*ΔpilY1.1 pVan-pilW1*(pEM668)	This study

References

1. Campos, J. M. & Zusman, D. R. Regulation of development in Myxococcus xanthus: effect of 3':5'-cyclic AMP, ADP, and nutrition. *Proc. Natl. Acad. Sci. U. S. A.* **72**, 518–522 (1975).
2. Kalos, M. & Zissler, J. Transposon tagging of genes for cell-cell interactions in Myxococcus xanthus. *Proc. Natl. Acad. Sci. U. S. A.* **87**, 8316–8320 (1990).
3. Vlamakis, H. C., Kirby, J. R. & Zusman, D. R. The Che4 pathway of Myxococcus xanthus regulates type IV pilus-mediated motility. *Mol. Microbiol.* **52**, 1799–1811 (2004).
4. Mercier, R. *et al.* The polar Ras-like GTPase MglA activates type IV pilus via SgmX to enable twitching motility in Myxococcus xanthus. *Proc. Natl. Acad. Sci. U. S. A.* **117**, 28366–28373 (2020).
