# Supplementary material for: The differential expression of PilY1 proteins by the HsfBA phosphorelay allows twitching motility in the absence of exopolysaccharides
Source: PLoS Genet. 2022 Apr 29;18(4):e1010188. doi: 10.1371/journal.pgen.1010188 (PMC9109919; doi:10.1371/journal.pgen.1010188)
Supplement: S3 Table — (PDF) [file pgen.1010188.s022.pdf]

Supplementary Table S3

Plasmid	Expression plasmid	Source
pEM487	pBJ114 with construct for *ΔepsW*	This study
pEM456	Originally pMAT1 with construct for *ΔpilT*	1
pEM538	pBJ114 with construct for *ΔpilB*	2
pEM584	pBJ114 with *hsfB*1 (sup4)*	This study
pEM585	pBJ114 with *hsfB*2 (sup7)*	This study
pEM586	pBJ114 with *pilW1* (sup9)*	This study
pEM587	pBJ114 with *hsfA* (sup9)*	This study
pEM588	pBJ114 with *pilY1.1* (sup10)*	This study
pEM589	pBJ114 with *pilY1.1* (sup11)*	This study
pEM590	pBJ114 with *pilY1.1* (sup12)*	This study
pEM604	pBJ114 with construct for *ΔpilY1.1*	This study
pEM605	pBJ114 with construct for *ΔhsfA*	This study
pEM606	pBJ114 with construct for *ΔhsfB*	This study
pEM607	pBJ114 with construct for *ΔhsfAB*	This study
pEM620	pBJ114 with construct for *ΔpilY1.2*	This study
pEM621	pBJ114 with construct for *ΔpilY1.3*	This study
pEM631	pBJ114 with construct for PilY1.3-FLAG	This study
pEM632	pBJ114 with construct for PilY1.2-FLAG	This study
pEM633	pBJ114 with construct for PilY1.1-FLAG	This study
pEM637	pBJ114 with construct for *pilY1.3Δvwa*	This study
pEM638	pBJ114 with construct for *pilY1.1Δduf*	This study
pEM645	pSWU19 with construct for $P_{pilA}PilA^{D71C}$	2
pEM650	pBJ114 with construct for PilY1.1^{D498A}	This study
pEM619	pETPhos for His$_6$-HsfA purification	This study
pEM640	pET28a for His$_6$-PilY1.1$^{23\text{-}525aa}$ purification	This study
pEM654	pET28a for His$_6$-PilY1.1$^{23\text{-}1419aa}$ purification	This study
pMR3690	Plasmid for vanillate inducible expression	3
pEM668	pMR3690 with *pilW1*	This study

References

1. Bulyha, I. *et al.* Regulation of the type IV pili molecular machine by dynamic localization of two motor proteins. *Mol. Microbiol.* **74**, 691–706 (2009).
2. Mercier, R. *et al.* The polar Ras-like GTPase MglA activates type IV pilus via SgmX to enable twitching motility in Myxococcus xanthus. *Proc. Natl. Acad. Sci. U. S. A.* **117**, 28366–28373 (2020).
3. Iniesta, A. A., García-Heras, F., Abellón-Ruiz, J., Gallego-García, A. & Elías-Arnanz, M. Two systems for conditional gene expression in Myxococcus xanthus inducible by isopropyl-β-D-thiogalactopyranoside or vanillate. *J. Bacteriol.* **194**, 5875–5885 (2012).
